# Partially Open HIV-1 Envelope Structures Exhibit Conformational Changes Relevant for Coreceptor Binding and Fusion

**DOI:** 10.1016/j.chom.2018.09.003

**Published:** 2018-10-10

**Authors:** Haoqing Wang, Christopher O. Barnes, Zhi Yang, Michel C. Nussenzweig, Pamela J. Bjorkman

**Affiliations:** 1 Division of Biology and Biological Engineering, California Institute of Technology, 1200 E. California Boulevard, Pasadena, CA 91125, USA; 2 Laboratory of Molecular Immunology and Howard Hughes Medical Institute, The Rockefeller University, New York, NY 10065, USA

**Keywords:** HIV-1 envelope, CD4, viral membrane fusion, cryoelectron microscopy

## Abstract

HIV-1 Env, a trimer of gp120-gp41 heterodimers, mediates membrane fusion after binding host receptor CD4. Receptor binding displaces V1V2 loops from Env's apex, allowing coreceptor binding and opening Env to enable gp41-mediated fusion. We present 3.54 Å and 4.06 Å cryoelectron microscopy structures of partially open soluble native-like Env trimers (SOSIPs) bound to CD4. One structure, a complex with a coreceptor-mimicking antibody that binds both CD4 and gp120, stabilizes the displaced V1V2 and reveals its structure. Comparing partially and fully open Envs with closed Envs shows that gp41 rearrangements are independent of the CD4-induced rearrangements that result in V1V2 displacement and formation of a 4-stranded bridging sheet. These findings suggest ordered conformational changes before coreceptor binding: (1) gp120 opening inducing side-chain rearrangements and a compact gp41 central helix conformation, and (2) 4-stranded bridging-sheet formation and V1V2 displacement. These analyses illuminate potential receptor-induced Env changes and inform design of therapeutics disrupting viral entry.

## Highlights

• Cryo-EM structures of HIV-1 Env trimer in CD4-bound, partially open conformation
• CD4-induced conformational changes of HIV-1 Env V1V2 region
• Mechanism of Env trimer opening and coreceptor binding site exposure
• Structural biology models for early stages of HIV-1 Env-mediated membrane fusion

## Graphical Abstract

**Figure:**
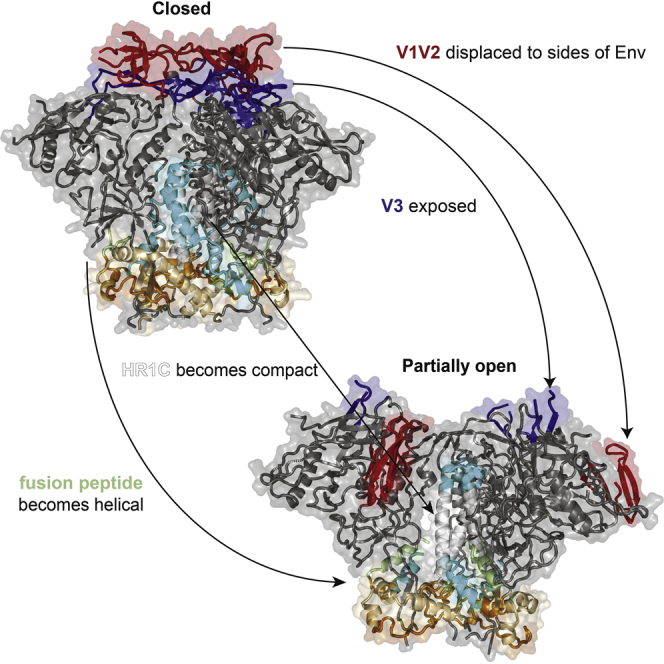

## Teaser

HIV-1 Env undergoes conformational changes after binding to host receptor CD4 that allow coreceptor binding, resulting in fusion between host and viral membranes. Wang, Barnes et al. solved cryo-EM structures of CD4-bound Envs, revealing the order of changes involved in coreceptor binding-site exposure and the structure of the rearranged V1V2 loops.

## Introduction

The first step in HIV-1 infection is fusion of the viral and host cell lipid bilayers to allow the HIV-1 capsid and its genetic material to enter the target cell (Harrison, 2015). Fusion is accomplished by HIV-1 Envelope (Env), a trimeric glycoprotein containing three copies of the receptor-binding gp120 subunit and three copies of the membrane-anchored gp41 subunit (West et al., 2014). Binding of gp120 to the host receptor CD4 induces conformational changes that expose the binding site for a coreceptor, CCR5 or CXCR4, whose binding results in further changes culminating in insertion of the gp41 fusion peptide into the host cell membrane and fusion of the two bilayers (Pancera et al., 2017).

Cryoelectron tomography/sub-tomogram averaging of Env trimers on HIV-1 virions revealed different conformational states, including unliganded Env in a closed, pre-fusion state with interactions across the gp120 trimer apex, and open CD4-bound Env that exhibited outwardly displaced and rotated gp120 subunits (Liu et al., 2008). The ∼20 Å resolution of these structures precluded detailed molecular interpretations, but higher resolution X-ray and single-particle cryoelectron microscopy (cryo-EM) structures of soluble native-like Env trimers (SOSIPs) (Sanders et al., 2013) in the closed, pre-fusion conformation were consistent with unliganded virion-bound cryoelectron tomography Env structures, and also revealed juxtaposition of the three gp120 V1V2 loop regions at the trimer apex that shield underlying regions of the coreceptor binding site on V3 (Ward and Wilson, 2017). An intermediate resolution (8.9 Å) single-particle cryo-EM structure of an SOSIP Env complexed with soluble CD4 (sCD4) and the coreceptor-mimicking antibody 17b (Sullivan et al., 1998) was consistent with the electron tomography structures of CD4-bound Env on virions (Liu et al., 2008) and showed ∼40 Å displacement of the V1V2 loops to the sides of the Env trimer to expose V3 (Wang et al., 2016a). These results were verified and extended in a 3.7 Å sCD4-and 17b-bound SOSIP structure, which also described side-chain rearrangements in gp120 and gp41 that were visible at higher resolution, but did not show ordered density for the rearranged V1V2 loops (Ozorowski et al., 2017).

We present two cryo-EM sCD4-bound SOSIP Env structures in complex with CD4-induced (CD4i) coreceptor-mimicking antibodies and with 8ANC195, a broadly neutralizing antibody (bNAb) that recognizes the gp120-gp41 interface (Scharf et al., 2014, Scharf et al., 2015, Scheid et al., 2011). The first structure, a complex of clade A/E BG505 SOSIP Env with sCD4 and Fabs from 17b and 8ANC195, was solved to a resolution of 3.54 Å (BG505-sCD4-17b-8ANC195 complex), allowing a detailed description of partial closure of the open sCD4-bound Env state that results from 8ANC195 binding. The second structure, solved at 4.06 Å resolution, is a complex of the clade B B41 SOSIP Env (Pugach et al., 2015) with sCD4, the CD4i antibody 21c (Xiang et al., 2002), and 8ANC195 (B41-sCD4-21c-8ANC195 complex). Despite binding of the 8ANC195 Fab that partially closes the open, sCD4-bound Env conformation, the structures show rearrangements in gp120, including displacement of V1V2, exposure of V3, formation of the 4-stranded bridging sheet, and formation of the α0 helix. In addition, unlike the V1V2 regions in the BG505-sCD4-17b-8ANC195 and B41-sCD4-17b structures, the displaced V1V2 loops in the B41-sCD4-21c-8ANC195 structure exhibited ordered density, allowing the structure of the displaced V1V2 to be determined. Comparisons of these partially open Env structures with fully open structures, including an open B41 Env complexed with b12 antibody against CD4-binding site (Ozorowski et al., 2017), showed that Env opening results in shifting of the gp41 helices to match post-fusion gp41 structures (Chan et al., 1997, Weissenhorn et al., 1997), and that Env opening and structural changes in gp120, such as V1V2 displacement and bridging-sheet formation, are independent steps. These comparisons suggest that Env opening in the absence of V1V2 displacement, a state that may exist in equilibrium with closed Env, triggers gp41 rearrangements as an initial step in the conformational changes required for fusion. A second, CD4-dependent step, involves V1V2 displacement and formation of the gp120 bridging sheet and α0 helix. Analyses of these results further our understanding of HIV-1 Env conformational changes leading to fusion and provide templates for designing agents to disrupt HIV-1 entry into target cells.

## Results

### Cryo-EM Structures of Envs Complexed with sCD4, Coreceptor-Mimicking Antibodies, and 8ANC195

EM studies confirmed that recombinant native-like soluble gp140 Env trimers (SOSIPs) (Sanders et al., 2013) can adopt the closed and open architectures seen on virion-bound Env trimers (Harris et al., 2011, Sanders et al., 2013, Tran et al., 2012). Thus, the SOSIP substitutions (SOS, a disulfide bond linking gp120 to gp41, and IP, an Ile→Pro mutation in gp41) do not prevent transition to the open Env state, although interpretations of sCD4-induced changes in gp41 must be interpreted cautiously since SOSIP Envs lack the membrane-proximal external regions, the transmembrane domains, and the cytoplasmic tails of gp41. For our cryo-EM structures, each sCD4-bound SOSIP Env was complexed with Fabs from a CD4i coreceptor-mimicking antibody (either 17b or 21c) (Sullivan et al., 1998, Xiang et al., 2002) and from 8ANC195, a gp120-gp41 interface bNAb (Scharf et al., 2014, Scharf et al., 2015, Scheid et al., 2011). CD4i Fabs were added to stabilize the coreceptor binding site and prevent Env closure because three CD4i Fabs cannot be accommodated on closed Env trimers due to steric clashes (Scharf et al., 2015). 8ANC195 Fab was added to rigidify the gp120-gp41 interface and induce partial closure of fully open, sCD4-bound Env (Scharf et al., 2015, Wang et al., 2016a), allowing investigation of the partially open Env conformation.

**Figure 1:**
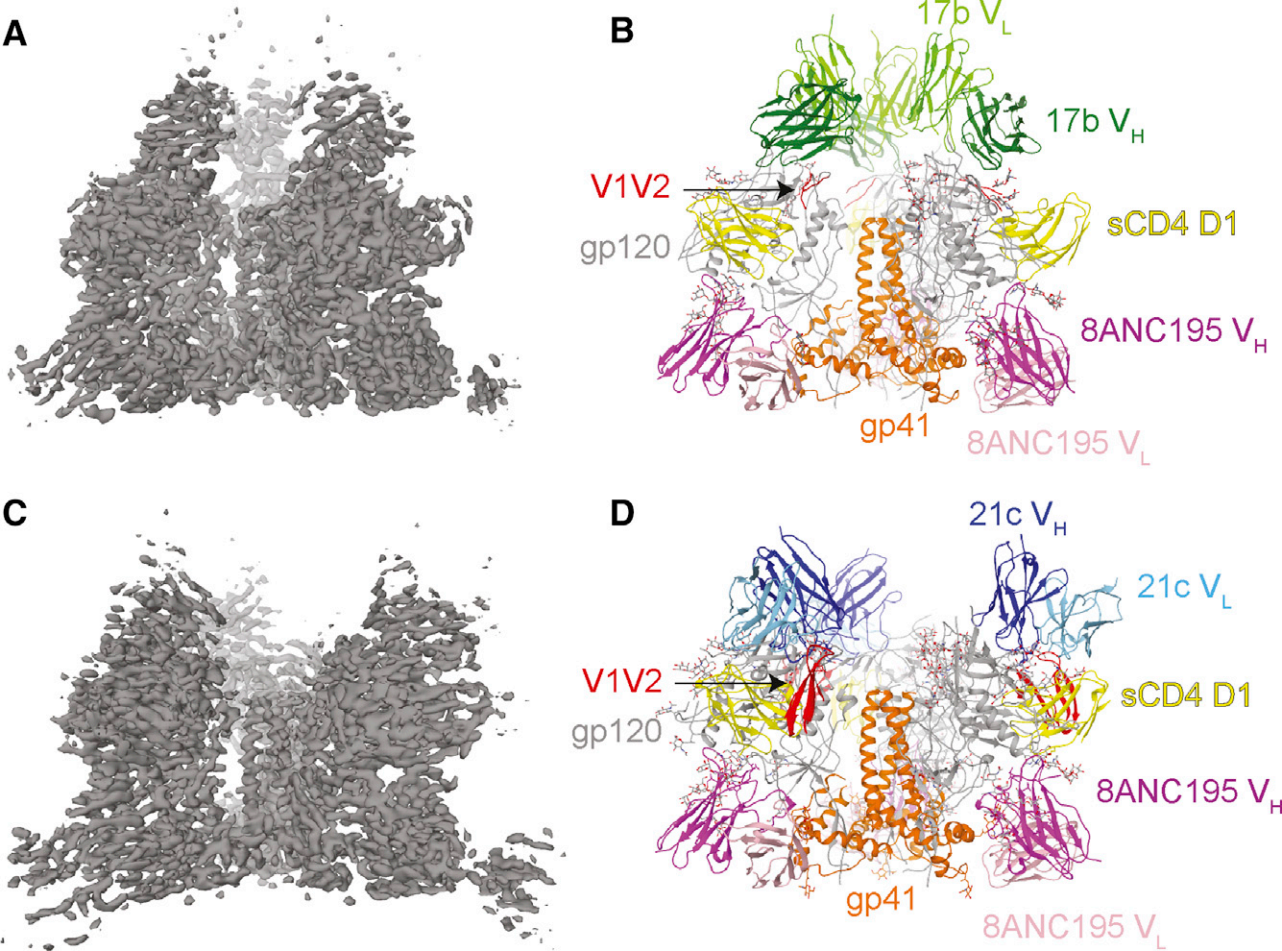
Cryo-EM Structures of sCD4-Bound Env Trimers (A) 3.54 Å density map of BG505-sCD4-17b-8ANC195 complex. (B) Fitted coordinates of BG505-sCD4-17b-8ANC195 complex. (C) 4.06 Å density map of B41-sCD4-21c-8ANC195 complex. (D) Fitted coordinates of B41-sCD4-21c-8ANC195 complex. The C_H_ and C_L_ domains of the Fabs and D2 domain of sCD4 were disordered in both structures and not fit into density. See also Tables S1 and S2 and Figures S1–S5.

**Figure 2:**
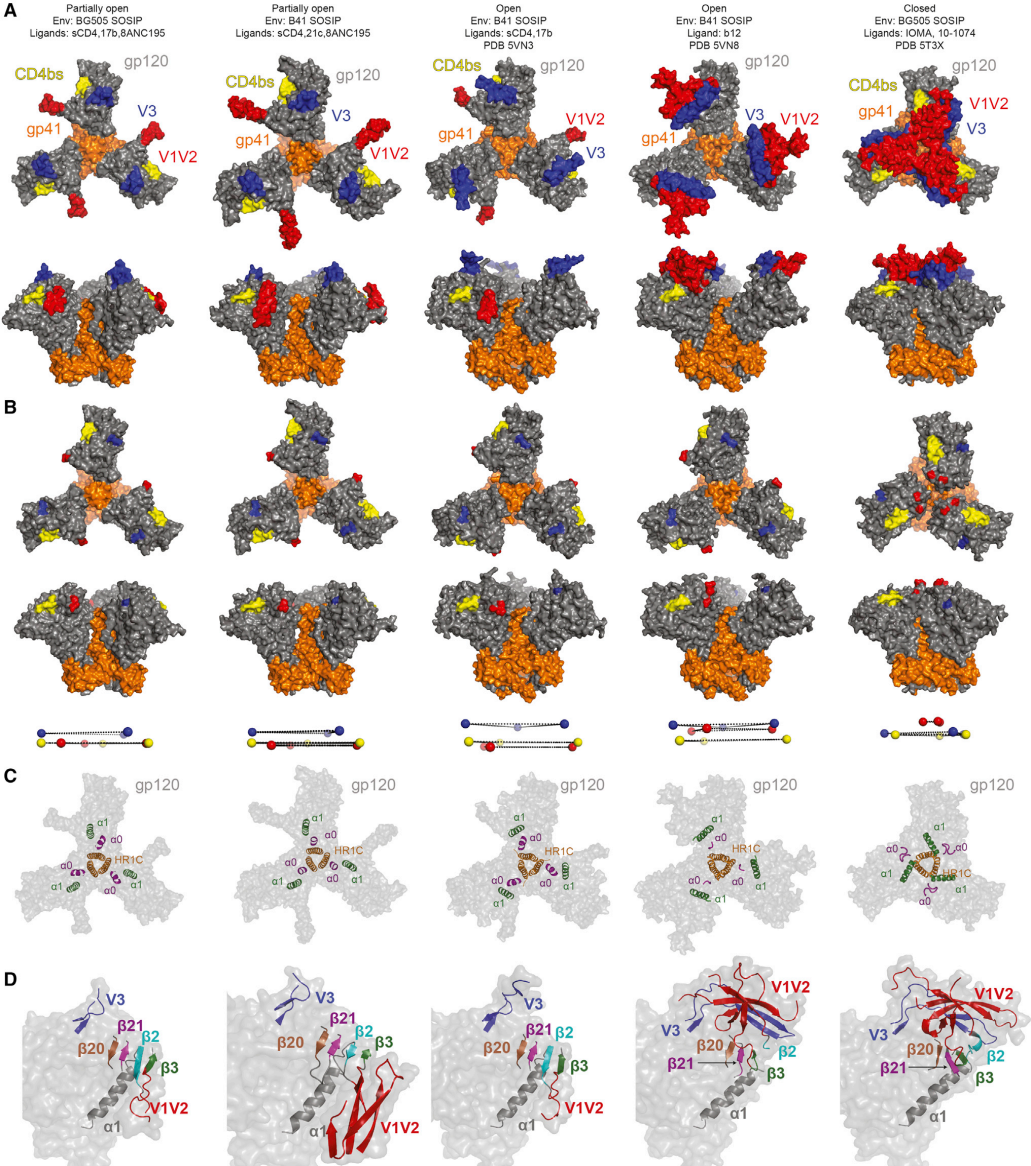
Comparison of Env Conformations (A) Top and side views of surface representations of Env trimers in partially open, sCD4-bound open, b12-bound open, and closed conformations. PDB: 5T3X is shown as a representative example of a closed Env structure; inter-protomer distances on closed Env structures are similar to each other despite strain-specific sequence differences and binding of different Fab ligands (Stadtmueller et al., 2018). (B) Structures shown in (A) with coordinates for residues 126–198 in V1V2 and 296–330 in V3 removed to facilitate seeing positions of landmark residues: CD4bs residues 364–372 (yellow), residue 125 and 199 at the base of V1V2 (red), and residues 295 and 331 at the base of V3 (blue). Positions of CD4bs residue 368, V1V2 base residue 124, and V3 base residue 330 are shown as spheres below their respective Env structures. (C) Conformational changes of α0. Structures in (A) and (B) are shown with coordinates for residues 64–73 in α0 and 98–117 in α1 in cartoon depiction overlaid on a transparent trimer surface. (D) gp120 monomers from structures shown in (A) and (B). β2, β3, β20, β21, V1V2 residues 126–198, and V3 residues 296–330 are shown in cartoon representations overlaid on a transparent gp120 surface. See also Table S3 and Figure S5.

Env-sCD4-Fab samples were prepared by adding 8ANC195 Fab to pre-formed open Env-sCD4-CD4i Fab complexes. Single-particle cryo-EM structures of BG505-sCD4-17b-8ANC195 and B41-sCD4-21c-8ANC195 complexes were solved to resolutions of 3.54 Å and 4.06 Å, respectively (Figures 1A–1D and S1–S4; Tables S1 and S2) (Env sequence alignments and definitions of secondary structural elements are shown in Figure S5). Each trimeric Env complex included three copies each of sCD4, CD4i Fab, and 8ANC195 Fab. The 17b and 21c Fabs exhibited distinct orientations relative to gp120s (Figures 1B and 1D), each consistent with crystal structures of these Fabs complexed with sCD4-bound gp120 cores (Diskin et al., 2010, Kwong et al., 1998). However, the BG505 and B41 Env trimers in our two structures were similar to each other (Table S3); thus, the binding of 17b versus 21c to the sCD4-bound Envs did not influence the extent of Env opening or the overall conformation of the Env trimers. As described in the 8.9 Å Env-sCD4-17b-8ANC195 structure (Wang et al., 2016a), the V1V2 loops were displaced from their location at the trimer apex to the sides of the trimer in the new partially open BG505 and B41 Env structures; however, portions of the displaced V1V2 were ordered in the B41-sCD4-21c-8ANC195 complex structure (Figure 1D). As seen in other sCD4-bound Env structures (Ozorowski et al., 2017, Wang et al., 2016a), the rearranged V3 loops were largely disordered in the two partially open sCD4-bound Envs (Figures 1B, 1D, and 2A).

### Comparison of Different Env Trimer Conformations

To evaluate conformational changes resulting from sCD4 and 8ANC195 Fab binding, we compared the partially open Env trimers with a 3.7 Å fully open B41 SOSIP in a B41-sCD4-17b complex (PDB 5VN3) (Ozorowski et al., 2017) and closed SOSIP Env structures (e.g., PDB: 5T3X) (Figure 2; Table S3). We also included a 3.6 Å cryo-EM structure of B41 Env complexed with the CD4-binding site bNAb b12 (PDB: 5VN8) in which the Env trimer exhibited an open conformation with respect to rotation and displacement of the gp120 subunits, but the V1V2 loops were not displaced to the sides of the Env trimer (Ozorowski et al., 2017).

We illustrated differences in gp120 positions in partially open, open, and closed Env trimers by measuring distances between gp120 landmarks (the CD4bs, the base of V1V2, and the base of V3) (Table S3) and highlighting their positions on Env structures (Figure 2A). To facilitate visualization of different gp120 orientations, we also highlighted the gp120 landmark residues on Env structures in which the coordinates for V1V2 and V3 residues were removed, demonstrating a progression of gp120 displacement relative to closed Env in partially open versus open Env structures (Figure 2B). The sCD4-bound Envs showed outward displacements and rotations of gp120s compared with closed Env, with the partially open sCD4-8ANC195-bound structures exhibiting smaller displacements from the closed Env conformation relative to the open, sCD4-bound Env conformation (Figure 2B; Table S3). The b12-bound open Env trimer also showed outward displacement of gp120s similar to the open sCD4-bound Envs (Figures 2A and 2B), but unlike the partially open and open sCD4-bound Envs in which the V1V2 loops were displaced to the side of Env trimer, the V1V2 loops remained “on top” of the displaced gp120s in b12-bound Env, where V1V2 was oriented equivalently with respect to the rest of gp120 as seen in closed Env structures (Figure 2D). Thus, opening of the b12-bound Env trimer involves a rigid body movement of the gp120-V1V2 unit such that the coreceptor binding site on the V3 loop remains largely buried, whereas the opening of gp120 subunits in sCD4-bound Envs includes repositioning of V1V2 to expose the V3 loop (Figure 2D). This suggests that Env trimer opening and CD4-induced coreceptor binding site exposure represent two distinct steps.

### Factors Contributing to Different Degrees of Env Trimer Openness

**Figure 3:**
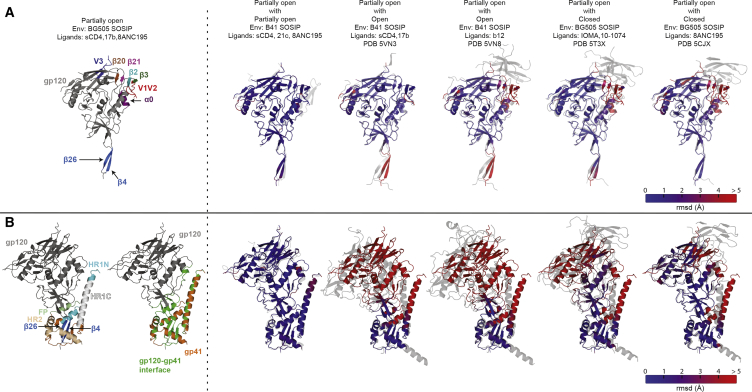
Conformations of the gp140 Protomer (A) Superimpositions of gp120 monomer from the BG505-sCD4-17b-8ANC195 Env trimer structure with gp120s from other Env trimer structures. Left: gp120 monomer from the BG505-sCD4-17b-8ANC195 structure with highlighted structural elements of interest. Right: gp120 from the BG505-sCD4-17b-8ANC195 structure (blue-red colors indicating RMSDs) superimposed with the indicated gp120s (gray). (B) Superimpositions of gp140 protomer from the BG505-sCD4-17b-8ANC195 Env trimer structure with gp140s from other Env trimer structures. Left: gp140 protomers from the BG505-sCD4-17b-8ANC195 structure with highlighted structural elements of interest (first structure) and highlighted gp120-gp41 interface regions (second structure). Right: gp140 from the BG505-sCD4-17b-8ANC195 structure (blue-red colors indicating RMSDs) superimposed with the indicated gp120s (gray). See also Figures S5–S7.

To evaluate differences in Env trimer openness, we aligned a gp120 monomer of our partially open BG505-sCD4-17b-8ANC195 structure with gp120 monomers from the partially open B41-sCD4-21c-8ANC195 structure, closed Env, and the two forms of open Envs. The gp120 subunits of partially open and fully open sCD4-bound Envs aligned with low root-mean-square deviation (RMSD) values in all regions except for the N- and C-terminal strands (β4 and β26), where the RMSDs increased to beyond 4 Å (Figure 3A). The β4 and β26 strands in the partially open sCD4-bound Envs were most similar in orientation to closed Envs, with closer agreement to an 8ANC195-bound closed Env structure than a closed Env solved in the absence of 8ANC195 (Figure 3A). Taken together, these results suggest that the β4 and β26 strands serve as a pivot point about which the rest of the gp120 subunit moves as a rigid body to either open Env trimer upon sCD4 or b12 binding or to partially close the sCD4-bound Env trimer conformation upon binding to 8ANC195.

We next investigated whether gp41 moves along with the gp120 β4 and β26 strands by aligning gp140s that were superimposed on their gp120-gp41 interface residues (defined as the gp120 and gp41 residues that include atoms within 5 Å of each other) (Figures 3B and S6). With the exception of interface residues on HR1 and the fusion peptide, the gp120-gp41 interfaces (highlighted in green in Figures 3B and S6) were mostly unchanged for different degrees of openness (Figure 3B). Thus, interactions across the gp120-gp41 interface in SOSIP Envs remain relatively unchanged during trimer opening. If this result extends to full-length Env trimers that do not contain the stabilizing SOSIP mutations, it suggests rigid body displacement of gp120 β4, β26, and the core portion of gp41 except for HR1C.

### gp41 Rearrangements

Each gp41 protomer in Env trimer comprises an N-terminal fusion peptide sequence, followed by two helical regions, HR1 and HR2 (Figure S5). The fusion peptides in the partially open SOSIP Env structures reside in a pocket at the gp120-gp41 interface as seen in the fully open Env conformation (Figure S7A). Although the ordered residues of the fusion peptide in closed and fully open Env structures form loops, the BG505-sCD4-17b-8ANC195 and B41-sCD4-21c-8ANC195 partially open Env structures include ordered fusion peptide residues in an α-helical conformation (Figures S4E, S4F, and S7A). The finding of helical fusion peptide residues likely represents the beginning of the formation of an energetically stable fusion peptide conformation that inserts into the target cell membrane during the fusion process (Harrison, 2015).

HR1C, the C-terminal portion of HR1, forms a parallel 3-helix bundle in Env trimers. The HR1C helical bundle in partially open Env gp41 subunits is extended at its N termini by ordered residues at the C termini of HR1N (N-terminal portion of HR1) to form longer and continuous α helices (Figure S7A). Extended HR1C helices are observed in fully open SOSIP Env structures, including the b12-bound open Env, but not in closed Envs (Figure S7A). In addition, the HR1C helical bundle is more compact in both partially open and fully open Envs than the 3-helical bundles in pre-fusion, closed Env structures (Figure S7A). Since the compact HR1C conformation was also observed in the B41-b12 structure (Ozorowski et al., 2017) (Figure S7A), these changes do not require V1V2 displacement. Notably, the compact HR1C conformation observed in all forms of open SOSIP Env matches the HR1C conformation seen in post-fusion gp41 structures in which HR1N forms a continuous α helix with HR1C (Chan et al., 1997, Weissenhorn et al., 1997) (Figures S7A and S7B). Taken together, these observations suggest that the change to a compact HR1C bundle that is extended at its N terminus with HR1N helical residues represents one of the first intermediate conformations in open structures on the path to the fusion-active conformation (Figure S7C). As these changes are also observed in the partially open Env trimers, 8ANC195 binding and partial closure of the open Env conformation does not reverse HR1C and HR1N changes induced by trimer opening.

### Internal gp120 Rearrangements

**Figure 4:**
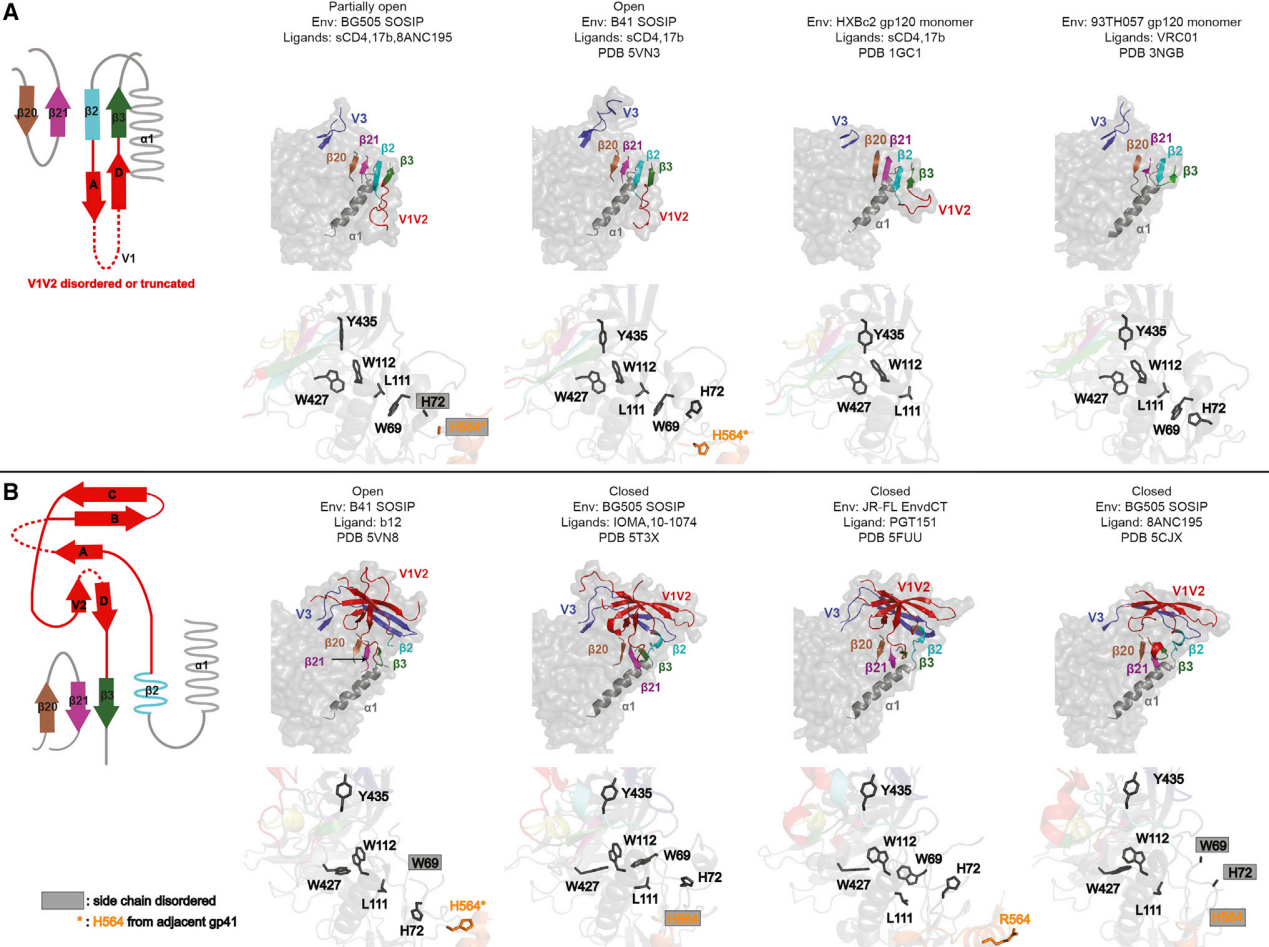
Network of Conserved Residues Regulating gp120 Conformational Changes (A) Bridging-sheet and conserved side-chain conformations in open Env trimer and gp120 monomer structures. Left: topology diagram of 4-stranded bridging sheet. Right: cartoon representations of β20, β21, β2, β3, α1, V1V2, and V3 overlaid on a transparent gp120 monomer surface (top) and stick representations of side chains of conserved residues of corresponding structures (bottom). (B) Bridging-sheet and conserved side-chain conformations in b12-bound open Env and closed Env trimer structures. Left: topology diagram of 3-stranded bridging sheet. Right: cartoon representations of β20, β21, β2, β3, α1, V1V2, and V3 overlaid on a transparent gp120 monomer surface (top) and stick representations of side chains of conserved residues of corresponding structures (bottom). See also Figure S4.

gp120 folding topologies are divided into two categories based on formation of the bridging sheet, an anti-parallel β sheet involving gp120 β strands β2, β3, β20, and β21 (Figure 4). The bridging sheet was first observed in structures of gp120 cores (gp120 monomers with truncations in V1V2, V3, and the N and C termini) in the presence and absence of sCD4 (Kwon et al., 2012, Kwong et al., 1998) (Figure 4A). However, the sheet is not fully formed in closed, pre-fusion Env trimers; instead of a 4-stranded anti-parallel β sheet, the β3, β20, and β21 strands form a mixed parallel and anti-parallel 3-stranded sheet, and the β2 residues adopt an α-helical conformation (Ward and Wilson, 2017) (Figure 4B).

The 4-stranded bridging sheet is found in both partially open sCD4-bound Env structures, with the rearranged V1V2 region located between β2 and β3 strands, similar to the fully open Env in the B41-sCD4-17b structure (Ozorowski et al., 2017). By contrast, the open Env in the B41-b12 complex adopts the 3-stranded β-sheet conformation of closed Env trimers (Figure 4B). Thus, the formation of the 4-stranded bridging sheet correlates with, and is likely required for, V1V2 displacement and exposure of the coreceptor binding site on V3. Also, in common with the open form of B41 bound to sCD4 and 17b (Ozorowski et al., 2017), the partially open BG505 and B41 Envs include the gp120 α0 helix, which interacts with the HR1 in the gp41 of an adjacent protomer, whereas the α0 region of the gp120s in the b12-bound open B41 Env and in closed Envs formed irregular loops (Figure 2C).

The B41-sCD4-17b structure revealed a series of gp120 side-chain rearrangements relative to closed Env structures; these side-chain rearrangements were not seen in the open B41-b12 complex (Ozorowski et al., 2017) (Figures 4A, 4B, and S4A), suggesting they are related to 4-stranded bridging-sheet formation induced by sCD4 binding rather than Env opening. A subset of the gp120 side-chain rearrangements (Tyr435_gp120_, Trp427_gp120_, Trp112_gp120_, Leu111_gp120_, and Trp69_gp120_) were observed in the 17b-bound partially open structure and monomeric gp120 structures (Figures 4A and S4A), demonstrating that (1) gp120 side-chain rearrangements occur both in BG505 and in B41 Envs upon binding to sCD4, (2) 8ANC195 binding and partial Env closure does not reverse these rearrangements, and (3) the rearrangements are related to 4-stranded bridging-sheet formation. The fact that the side-chain positions found in sCD4-bound Env trimers are also found in monomeric gp120 core structures, whether or not they are complexed with sCD4, indicates that this collection of side-chain conformations is related to formation of the 4-stranded bridging sheet (Figure 4A).

### Structure of Displaced V1V2

**Figure 5:**
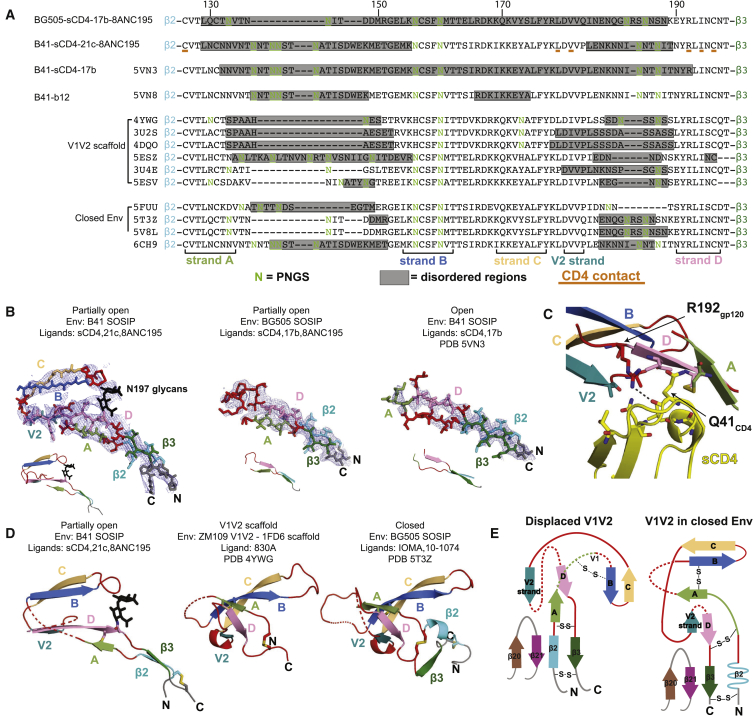
V1V2 Conformations in Different Structures (A) Sequence alignments of the V1V2 region from representative Env structures, including different conformations of Env trimers and monomeric V1V2 scaffolds. The A–D and V2 strands are indicated. Disordered regions in each structure are indicated by a gray background. Potential N-linked glycosylation sites (PNGSs) are highlighted in green and contacts with sCD4 (defined as residues in V1V2 with an atom within 5 Å of an sCD4 residue) in the B41-sCD4-21c-8ANC195 structure are underlined in red. (B) Models of displaced V1V2 residues (stick representation) with accompanying density contoured at 8σ (0.047 e/Å^3^) for the indicated structures. Insets: cartoon representations of the V1V2 models. (C) Close up of V1V2 interactions with sCD4 (yellow) in the B41-sCD4-21c-8ANC195 structure. A potential interaction with V1V2 residue Arg192_gp120_ and sCD4 is denoted as a black dashed line, and was identified based on distance. (D) Cartoon representations of V1V2 regions in the indicated structures. Disordered regions are indicated by dashed lines. (E) Topology diagrams of V1V2 structures shown in (D).

**Figure 6:**
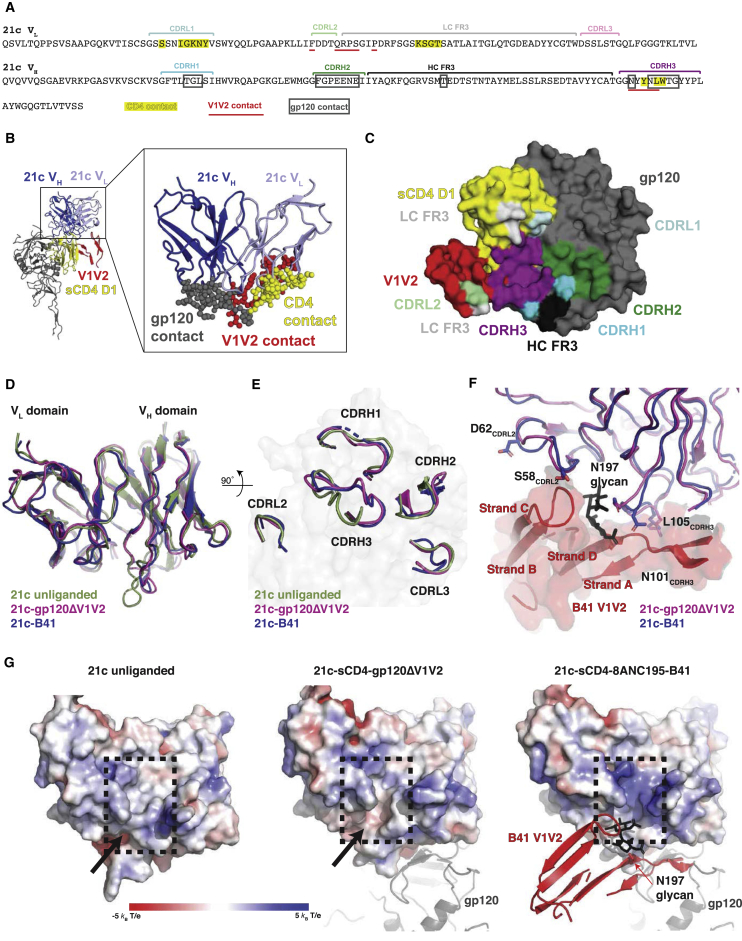
21c Epitope on gp120 and sCD4 (A) Sequence of 21c V_H_V_L_ with CDR residues indicated by brackets, and sCD4-contacting residues highlighted in yellow, V1V2-contacting residues in red, and gp120-contacting residues in gray. Contacting residues in the 21c paratope and epitope were defined as two residues containing an atom within 5 Å of each other. (B) Structure of a 21c, sCD4, gp120 protomer in the B41-sCD4-21c-8ANC195 structure, with inset showing residues contacting V1V2, sCD4, and gp120 highlighted. (C) 21c epitope on a gp120-sCD4 complex (surface representation seen from the top). Highlighted regions of the sCD4 and gp120 surfaces are contacted by the indicated regions of 21c. To prevent overinterpretation of contacts at low resolution, the displaced V1V2 was modeled as polyalanine unless side-chain density at ≥7σ (0.04 e/Å^3^) was observed. (D) Superimposition of V_H_V_L_ domains from structures of unliganded 21c Fab (PDB: 3LMJ; green), 21c Fab bound to a sCD4 complex with monomeric gp120ΔV1V2 core (PDB: 3LQA; magenta), and 21c from the B41-sCD4-21c-8ANC195 complex (this study; blue). (E) View of CDRs in the superimposition shown in (D). (F) Close up of 21c interaction with V1V2 and Asn197_gp120_ glycan. (G) Comparison of electrostatic surface potentials (positive potential shown as blue, negative potential shown as red) of unliganded 21c (left), sCD4 plus gp120 core-bound 21c (middle), and B41 Env trimer plus sCD4-bound 21c (right) showing the opening of a positively charged cleft (black arrow and box) that could accommodate negatively charged terminal sialic acids on the complex-type Asn197_gp120_ glycan on V1V2 (right panel; black sticks).

The V1V2 region comprises a twisted 5-strand β sheet (strands A–D and a V2 strand) with mostly disordered connecting loops in monomeric epitope scaffolds (Gorman et al., 2016, McLellan et al., 2011, Pan et al., 2015, Pancera et al., 2013), closed Envs (Ward and Wilson, 2017), and the b12-bound open Env (Ozorowski et al., 2017) (Figure 5A). V1V2s interact across the trimer apex in closed Envs to shield the underlying coreceptor binding sites on the V3 regions (Ward and Wilson, 2017) (Figure 2A) and are displaced by ∼40 Å to the sides of Env upon sCD4 binding (Wang et al., 2016a). Density observed at low contour levels for the displaced V1V2 regions in our 8.9 Å cryo-EM structure of BG505-sCD4-17b-8ANC195 was uninterpretable (Wang et al., 2016a), and the displaced V1V2 regions were disordered in our 3.54 Å structure of this complex and in the 3.7 Å B41-sCD4-17b structure (Ozorowski et al., 2017) (Figures 5A and 5B). However, we found interpretable density for portions of the displaced V1V2 in the B41-sCD4-21c-8ANC195 structure (Figure 5B), likely because 21c, unlike 17b, interacts with V1V2 (Diskin et al., 2010) (Figure 6) to stabilize a conformation that conserves V1V2 structural elements (Figure 5A).

The density for the displaced V1V2 in the B41-sCD4-21c-8ANC195 structure, although at lower resolution than central parts of the complex (Figure S3D), showed interactions with sCD4 (Figure 5C) and could be modeled as five β-strands using information from structures of V1V2 in monomeric scaffolds and closed Env trimers (Figure 5D). Our model for the displaced V1V2 density preserves the strand B-strand C and strand A–D interactions observed in the V1V2 regions in closed Env and V1V2 scaffold structures (Figure 5D) and predicted in molecular dynamics simulations of the displaced V1V2 structure (Yokoyama et al., 2016). In our model, strands A and D continue the anti-parallel β sheet that starts and ends with the gp120 β2 and β3 strands in Env structures with a 4-stranded bridging sheet (Figures 5D and 5E). Strand A continues into strand B through a loop that varies in length across Env strains and is disordered in most V1V2 scaffolds and closed Env trimer structures (Figure 5A). The loop connecting strands B and C, highly conserved across HIV-1 strains (Los Alamos HIV Sequence Database; www.hiv.lanl.gov/) interacts with CDRL2 and CDRH3 of 21c (Figures 6A–6C). Strand C is connected through a loop to the V2 strand, which is then joined to the N-terminal portion of strand D through a disordered loop. Whereas the V2 and D strands are anti-parallel in V1V2 scaffold and closed Env structures, V2 appears to be parallel to strand D in the displaced V1V2 (Figures 5D and 5E) based on density for the strand C to V2 connection. In addition, the short length of the strand C to V2 connection (two residues) would not permit placement of the V2 strand in an anti-parallel orientation relative to strand D. The change to create a parallel V2 strand-D strand interaction could result from the rearrangement of the β2 and β3 regions that occurs in the switch from the 3-stranded bridging-sheet conformation in closed Env to the 4-stranded bridging sheet in sCD4-bound Envs (Figure 5E). Contacts between the displaced V1V2 and sCD4 predicted from the 8.9 Å structure of BG505-sCD4-17b-8ANC105 (Wang et al., 2016a) were revealed at higher resolution in the B41-sCD4-21c-8ANC195 structure to involve interactions between sCD4 strands B, E, and the D–E connecting loop with the V1V2 D and V2 strands (Figures 5A and 5C).

### 21c Epitope on CD4 and gp120

The epitope of 21c has been structurally characterized as including both a foreign antigen (gp120) and a self-antigen (CD4); indeed, it is the only HIV-1 CD4i antibody described to bind sCD4 weakly in the absence of gp120 (Diskin et al., 2010). A structure of the clade C CAP210 gp120 monomeric core complexed with 21c and sCD4 revealed gp120 and sCD4 contacts with 21c, but potential interactions with V1V2 could not be visualized because the V1V2 region was truncated in the core gp120 construct (Diskin et al., 2010). In the B41-sCD4-21c-8ANC195 structure, 21c interactions with the core region of gp120 map exclusively to the heavy chain, and interactions with sCD4 map primarily to the light chain (Figure 6A), conserving critical interactions observed in the gp120-21c-sCD4 crystal structure despite gp120 sequence variations. In addition, density revealed that the 21c light chain engages directly with the loop connecting strands B and C of the displaced V1V2 (Figures 6A and 6F), consistent with binding results that showed an increase in 21c affinity for V1V2-including gp120s (Diskin et al., 2010).

To ascertain the effects of the 21c interactions with V1V2, we compared the structures of the 21c Fab in its free, monomeric core gp120-, and B41-bound forms by superimposing the variable domains (Figures 6D and 6E). These comparisons revealed that 21c undergoes binding-induced conformational changes in the presence of gp120 and sCD4, with the largest change observed in the CDRH3 loop (RMSD = 2.4 Å for superposition of 16 CDRH3 Cαs in free and B41-bound 21c) (Figures 6D and 6E). Comparison of the complexed 21c Fabs revealed minor changes in variable domain conformations (RMSD = 0.9 Å for superposition of 235 21c V_H_-V_L_ Cαs in the gp120-sCD4-21c and B41-sCD4-21c-8ANC195 structures), associated with the presence of the displaced V1V2 and a complex-type N-glycan attached to Asn197_gp120_ in the B41 Env trimer (Cao et al., 2017) that was not present in the gp120 core used for the gp120-sCD4-21c structure (Figure 6F).

Interestingly, CDRH3 conformational changes observed in complexed 21c structures not only allow for 21c residues Leu105_HC_ and Glu55_HC_ to insert into pockets on gp120 (Diskin et al., 2010) (Figure 6F) but also promote the formation of a groove comprising CDRH3, CDRL2, and FRL2, which is likely to accommodate the Asn197_gp120_ glycan that is observed in >98% of HIV-1 strains (Figure 6G) (Los Alamos HIV Sequence Database; https://www.hiv.lanl.gov/). Furthermore, binding-induced conformational changes resulting in a more electropositive groove in bound 21c compared with the free antibody suggests interactions with negatively charged sialic acids on the complex-type glycan attached to Asn197_gp120_ (Behrens et al., 2016, Cao et al., 2017, Gristick et al., 2016).

## Discussion

**Figure 7:**
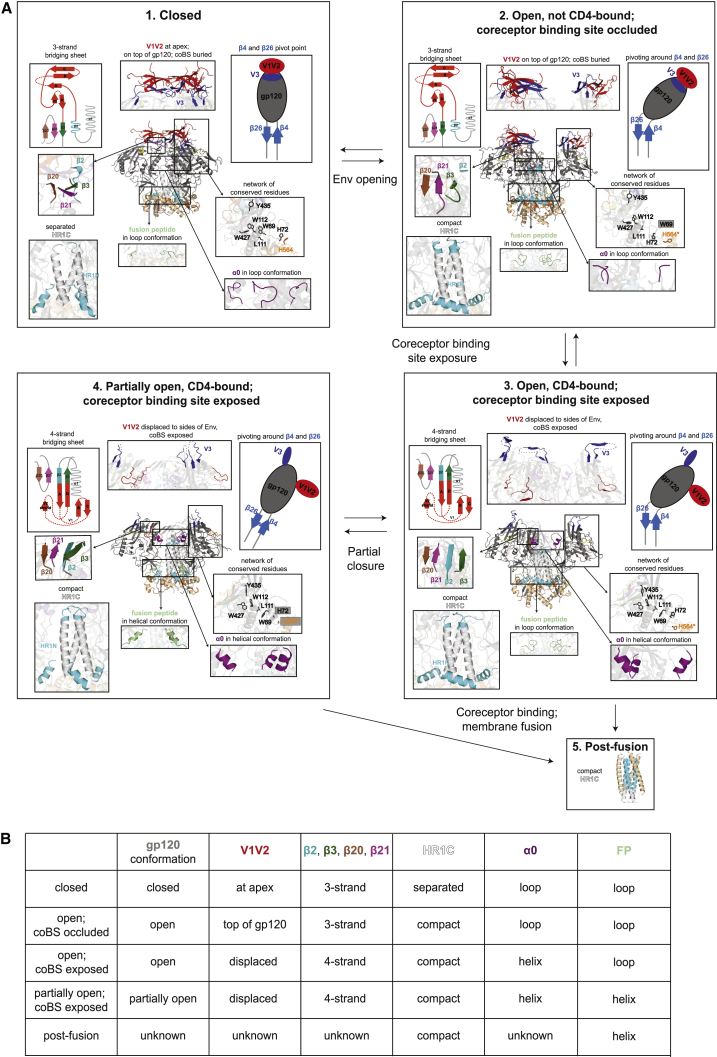
Model for Order of Conformational Changes Leading to Coreceptor Binding and Fusion (A) Overview of features in different HIV-1 Env trimer conformational states. Five conformations that have been characterized by X-ray crystallography or cryo-EM are listed with their corresponding structural features. (1) PDB: 5T3Z; (2) PDB: 5VN8; (3) PDB: 5VN3; (4) this study; (5) PDB: 1AIK. (B) Table summarizing structural features of the conformational states listed in (A). coBS, coreceptor binding site; FP, fusion peptide. See also Video S1.

The HIV-1 Env glycoprotein must adopt multiple conformations to function in fusion of the viral and host cell membranes (Ward and Wilson, 2017). Although we cannot define mechanisms for structural transitions from static structures that, by necessity, contain bound ligands and antibodies, by comparing our cryo-EM structures of sCD4-bound partially open SOSIP Env trimers with structures of Env in other conformational states, we reveal information about how Env trimers rearrange to expose the coreceptor binding site and describe which parts of the rearrangement are reversible upon binding to a gp120-gp41 interface bNAb (Video S1). In particular, we captured an ordered conformation of the displaced V1V2 loops that move from the trimer apex to the sides of open Env upon sCD4 binding. We also showed that the structural rearrangements of extending and compacting the gp41 central helices are an initial step to achieving an open Env conformation (Figure 7) in the SOSIP Env trimers being compared.

Video S1. Conformational Changes of HIV-1 Env Trimer, Related to Figure 7

With respect to the coreceptor binding site, the most relevant feature of the closed Env trimer structure is the location of the V1V2 loops at the trimer apex that shield the coreceptor binding site on V3 (Figure 7A, panel 1). The apex location of the V1V2 loops and their burial of V3 requires the 3-stranded form of the gp120 bridging sheet. The HR1C region of the three central gp41 helices are relatively separated from each other in the closed conformation, and the α0 region adjacent to HR1C adopts a loop conformation (Figure 7A1).

We hypothesize that a later, but still early, conformation on the pathway toward coreceptor binding site exposure is represented by the b12-bound open conformation of the B41 HIV-1 Env (Figure 7A2) (Ozorowski et al., 2017). In this structure, the gp120 subunits have rotated outward from the trimer axis. However, although the Env trimer is open, the V1V2 loops are not displaced from their location at the top of each gp120 subunit, thus leaving the V3 loop partially buried by V1V2 (Figure 7A2). It is unclear if the b12 antibody captures a pre-existing open Env state that is in equilibrium with closed Env; if so, this state is not detectable in double electron-electron resonance spectroscopy analyses of SOSIP Env conformations (Stadtmueller et al., 2018). Thus, the b12-bound open state could either be induced by b12 binding or exist at levels that are spectroscopically undetectable but that can nevertheless can be captured by b12. This state may represent an early intermediate after sCD4 binding to the closed, pre-fusion state, in which gp120 rotation is a prerequisite to V1V2 displacement (Figure 7A3).

The B41-b12 structure demonstrates that Env trimer can open even without forming the 4-stranded gp120 bridging sheet, likely based on gp41 rearrangements and rotation about the pivot point defined by the gp120 β4 and β26 strands. Moreover, gp120 opening in the absence of 4-stranded bridging-sheet formation triggers some of the structural rearrangements seen in sCD4-bound structures; for example, some conserved gp120 side chains (Trp69_gp120_, His72_gp120_, Leu111_gp120_) are rearranged with respect to the closed structure (Figures 7A1 and 7A2). Interestingly, the HR1C helices adopt the compact conformation found in post-fusion gp41 structures (Chan et al., 1997, Weissenhorn et al., 1997) and also include HR1N residues as part of a longer central gp41 helical region.

The final conformation of open Env on the pathway to coreceptor binding is observed in the sCD4-bound Env structures in which the gp120 subunits have opened, as in the b12-bound conformation, but the V1V2 loops have also been displaced to the sides of Env trimer to expose the V3 loop for interactions with coreceptor (Figure 7A3). However, the V3 residues are largely disordered in this structure and in the partially open Env structures with displaced V1V2 loops, suggesting that flexibility in V3 facilitates interactions with coreceptor. In coreceptor binding-site-exposed open conformations, the gp120 bridging sheet adopts a 4-stranded conformation in which the β2 residues form a β-strand connected to the N-terminal strand A of V1V2. The β2 and β3 gp120 elements also swap positions so that the four strands of the bridging sheet are now anti-parallel, allowing the displacement of V1V2 from the top of each gp120 to its side, thereby exposing V3. The HR1C helices in gp41 retain their compact configuration with extended helical regions from HR1N residues at their N termini. Some conserved gp120 residues adopt altered side-chain conformations that were proposed to allosterically transfer the signal from the sCD4 binding site to the gp120-gp41 interface, such that the structure of gp41 is altered (Ozorowski et al., 2017). For example, the gp120 α0 residues are now in a helical conformation that tops the HR1C from a neighboring gp41protomer.

The open, coreceptor binding-site-exposed conformation goes on to bind CCR5 or CCR4, adopting uncharacterized fusion-active conformation(s) that promotes fusion of the viral and host cell membranes. The only available structures of a conformational state that follows the open, coreceptor binding-site-exposed conformation are structures of 6-helical bundles of HR1 and HR2 portions of gp41 (Chan et al., 1997, Weissenhorn et al., 1997) (Figure 7A5), which presumably represent post-fusion conformations of gp41. These structures contain the continuous HR1N-HR1C helix in a compact 3-helix bundle, which is similar to open, but not closed, Env trimer structures.

Having categorized features of available Env structures in terms of their relationship to each other and to the post-fusion gp41 conformation, our structures address the pliability of the sCD4-bound conformation by showing that the sCD4-bound open conformation can partially close through binding to 8ANC195 (Video S1), a gp120-gp41 interface bNAb that also binds closed Envs (Scharf et al., 2015). In our two Env-sCD4-8ANC195 structures, the gp120 subunits are not as separated from each other as in fully open conformations, therefore we refer to this conformation as partially open (Figure 7A4). The complexes for these structure determinations were formed by incubating fully open, sCD4-bound Env trimers with 8ANC195, suggesting that 8ANC195 either captured a partially open, sCD4-bound conformation or its binding triggered partial closure of the open conformational state. Whether the partially open sCD4-bound conformation of Env trimer represents a dead-end conformation of Env or a coreceptor binding site-exposed conformation of Env that could proceed along the pathway to viral/host cell membrane fusion is unknown. In any case, the structural comparisons show that partial closure of the sCD4-bound open state does not reverse the formation of 4-strand bridging sheet, V1V2 displacement, coreceptor binding site exposure, α0 helix conformation, or the formation of compact gp41 HR1C helices with HR1N extensions (Figure 7A4; Video S1). However, not all of the gp120 side-chain rearrangements are conserved with the fully open sCD4-bound structure. Interestingly, an ordered portion of the gp41 fusion peptide, which adopts a loop conformation in closed and other open Env structures, is α-helical in both partially open Env structures. As the fusion peptide is thought to insert into the target membrane as a helix (Harrison, 2015), the partially open structures show a propensity of the fusion peptide to shift from a loop into a helix in preparation for mediating fusion of the viral and host membranes.

Although disordered in other sCD4-bound complexes, the conformation of the displaced V1V2 region was revealed in the B41-sCD4-21c-8ANC195 complex. The secondary structural elements of V1V2 and regions of flexibility indicated by disorder are mostly maintained whether V1V2 is displaced to the sides of Env trimer, interacts about the apex of a closed Env trimer, or is expressed as a monomeric V1V2 scaffold. This suggests that, in the CD4-bound conformation, the V1V2 secondary structural elements move as a rigid body about the loops extending from strands A and D. Thus, the B41-sCD4-21c-8ANC195 structure likely reveals its architecture because of the stabilizing effects of 21c via interactions with the B-C loop region, while in the 17b-bound open structures, the displaced V1V2 occupies multiple positions that are averaged out during map reconstruction.

Comparisons of available Env structures provide clues as to what controls displacement of V1V2 to expose the coreceptor binding site in sCD4-bound structures. We note that in all Env structures with a 4-stranded bridging sheet, including Env trimers in fully and partially open conformations, as well as gp120 monomer structures plus and minus bound sCD4, the side-chain conformations of conserved gp120 residues Tyr435_gp120_, Trp427_gp120_, Trp112_gp120_, Leu111_gp120_, and Trp69_gp120_ are consistent. By contrast, in all structures with 3-stranded bridging sheets (closed Env trimers and b12-bound open Env), the conserved residues adopt distinct conformations. These results suggest that the side-chain rearrangements in these residues control the reorganization of the β2, β3, β20, and β21 structural elements and thus regulate 4-stranded bridging-sheet formation and coreceptor binding-site exposure. Thus, structures of monomeric gp120 cores, which include a 4-stranded bridging sheet, mimic the sCD4-bound and coreceptor binding-site-exposed conformation of gp120 in Env trimers, rather than the conformation in closed, pre-fusion Env trimers.

Many structural questions related to Env conformations remain to be elucidated, most notably the fusion-active conformation that results from coreceptor binding. However, the structures reported here provide critical information for understanding steps leading up to coreceptor binding-site exposure, including (1) suggesting an order for Env conformational changes, (2) describing elements of conformational plasticity in the sCD4-bound Env state, and (3) revealing a structure for the V1V2 regions displaced by sCD4 binding. This information adds mechanistic details to Env-mediated membrane fusion and provides a more complete sCD4-bound structure that serves as a template for understanding non-neutralizing, and potentially neutralizing, antibody responses to open Env conformations.

## STAR★Methods

### Key Resources Table

**Table:** 

REAGENT or RESOURCE	SOURCE	IDENTIFIER
**Chemicals, Peptides, and Recombinant Proteins**		

tris(2-carboxyethyl)phosphine (TCEP)	Pierce	Cat#20491
bis(2,2,5,5-tetramethyl-3-imidazoline-1-oxyl-4-il)-disulfide	Enzo	Cat# ALX-430-102-M010
Papain	Pierce	Cat#20341
HEPES	Thermo Fisher Scientific	Cat#15630080
MEM Non-Essential Amino Acid Solution (MEM NEAA)	Thermo Fisher Scientific	Cat#11140050
GlutaMAX Supplement	Thermo Fisher Scientific	Cat#35050061
Sodium Pyruvate	Thermo Fisher Scientific	Cat#11360070
Hygromycin B	Thermo Fisher Scientific	Cat#10687010
BG505 SOSIP.664 v3.2	(Sanders et al., 2013)	N/A
B41 SOSIP.664 v 4.2	(Pugach et al., 2015)	N/A
17b Fab	(Thali et al., 1993)	N/A
2G12 IgG	(Buchacher et al., 1994)	N/A
21c IgG	(West et al., 2010, Xiang et al., 2002)	N/A
sCD4	(Brighty et al., 1991)	N/A
b12 Fab	(Burton et al., 1991)	N/A
8ANC195 Fab	(Scharf et al., 2014)	N/A

**Critical Commercial Assays**		

FreeStyle 293 Expression Medium	Thermo Fisher Scientific	Cat#12338018
ProCHO-5 serum-free medium	Sartorius	Cat#BE12-766Q

**Deposited Data**		

BG505-sCD4-17b-8ANC195 cryo-EM map	EMDB	EMDB: 7516
BG505-sCD4-17b-8ANC195 coordinates	PDB	PDB: 6CM3
B41-sCD4-21c-8ANC195 cryo-EM map	EMDB	EMDB: 9038
B41-sCD4-21c-8ANC195 coordinates	PDB	PDB: 6EDU

**Experimental Models: Cell Lines**		

HEK293-6E	National Research Council of Canada	Cat#11565
CHO Flp-In cells	Invitrogen	Cat# R75807

**Recombinant DNA**		

pTT5 mammalian expression vector (used to express all BG505 SOSIP variants and all ligands)	National Research Council of Canada	N/A

**Software and Algorithms**		

Pymol	Schrödinger LLC; https://www.schrodinger.com/pymol	RRID: SCR_000305
UCSF Chimera	(Pettersen et al., 2004)	RRID: SCR_004097
Phenix	(Adams et al., 2010)	RRID: SCR_014224
Coot	(Emsley and Cowtan, 2004)	RRID: SCR_014222
Relion	(Scheres, 2012)	RRID: SCR_016274
SerialEM	(Mastronarde, 2005)	http://bio3d.colorado.edu/SerialEM/
Rosetta	(Wang et al., 2016b)	RRID: SCR_015701

**Other**		

HiLoad 16/600 Superdex 200 pg column	GE Healthcare	Cat#28989335
HiTrap Q HP, 5 mL column	GE Healthcare	Cat#17115401
Superose 6 10/300 GL column	GE Healthcare	Cat#17517201
2G12 5 ml column made in-house using using NHS-activated HP resin and 2G12 IgG	GE Healthcare	Cat#17071601
Protein A column	GE Healthcare	Cat#17040301
400 Mesh Quantifoil R1.2/1.3 copper grids	EM Resolutions	QR1213400Cu50
300 Mesh Quantifoil R1.2/1.3 gold grids	EM Resolutions	QR1213400Au50

### Contact for Reagent and Resource Sharing

Further information and requests for resources and reagents should be directed to and will be fulfilled by the Lead Contact, Pamela J. Bjorkman (bjorkman@caltech.edu). The Bjorkman laboratory cannot lawfully distribute clones in the pTT5 vector. Those wishing to obtain these clones must first obtain a license from the National Research Council of Canada (see Key Resources Table).

### Experimental Model and Subject Details

#### Cells Lines

HEK293-6E (female) and CHO Flp-In (female) cell lines were stored at liquid nitrogen temperature. Before transfection, HEK293-6E cells were maintained in FreeStyle 293 Expression Medium, CHO Flp-In cells were maintained in ProCHO5 medium supplemented with HEPES, GlutaMAX, MEM NEAA, Sodium Pyruvate, and Hygromycin B. Cells were maintained at 37°C in a humidified shaker at 5% CO_2_.

### Method Details

#### Protein Expression and Purification

His_6_-tagged Fabs from 17b, and the 8ANC195_G52K5_ variant of 8ANC195 were expressed by transient transfection in HEK293-6E cells (National Research Council of Canada) and purified from cell supernatants using Ni-NTA chromatography and size exclusion chromatography (SEC) as described (Scharf et al., 2015). The heavy and light chain genes encoding 21c IgG were isolated as described (West et al., 2010) from an Epstein Barr virus-transformed human B-cell line (Xiang et al., 2002) obtained from James Robinson (Tulane University), and 21c IgG was expressed by transient transfection in HEK 293-6E cells. 21c IgG was isolated from transfected cell supernatants by Protein A chromatography (GE Healthcare), and 21c Fab was obtained by digesting with immobilized papain (Pierce) at 10 mg ml^−1^ and purified by protein A (GE Healthcare) and SEC chromatography as described (Diskin et al., 2010). His_6_-tagged sCD4 D1D2 (domains 1 and 2; residues 1–186 of mature CD4) was expressed by transient transfection in HEK293-6E cells and purified by Ni-NTA chromatography and SEC.

BG505 SOSIP.664 v3.2 (in vector pTT5, National Research Council of Canada), a native-like soluble gp140 trimer (Sanders et al., 2013) including the ‘SOS’ substitutions (A501C_gp120_, T605C_gp41_), the ‘IP’ substitution (I559P_gp41_), the *N*-linked glycan sequence at residue 332_gp120_ (T332N_gp120_), an enhanced gp120-gp41 cleavage site (REKR to RRRRRR), and a stop codon after residue 664_gp41_ (Env numbering according to HX nomenclature) was expressed in HEK293-6E cells in the absence of kifunensine and was purified from cell supernatants by 2G12 immunoaffinity chromatography and SEC as previously described (Wang et al., 2017). B41 SOSIP.664 v4.2 (Pugach et al., 2015) was expressed in CHO Flp-In cells (Invitrogen) using vector pIPP4 (Chung et al., 2014) using cell lines kindly provided by Al Cupo and John Moore (Weill Cornell Medical College) and purified as described for BG505. All proteins were stored at 4°C in 20 mM Tris, pH 8.0, and 150 mM sodium chloride (TBS buffer) supplemented with 0.02% (w/v) sodium azide.

#### Sample Preparation

BG505-sCD4-17b-8ANC195 and B41-sCD4-21c-8ANC195 complexes were prepared by incubating purified Env with sCD4 at a 1:3 molar ratio (gp140 protomer:sCD4) for 4 h at room temperature, followed by subsequent incubation with a 1:3 molar ratio of CD4i Fab (gp140 protomer:17b or 21c) overnight at 4°C. Complexes were isolated by SEC in TBS (20 mM Tris pH 8.0, 100mM NaCl) using a Superose 6 10/300 column (GE Healthcare) and peak fractions analyzed by SDS-PAGE. Fractions corresponding to the Env-sCD4-CD4i complex were pooled and a 10-fold molar excess of 8ANC195 Fab was added before incubation at room temperature for 2 h. Complexes were again isolated by SEC using a Superose 6 10/300 column (GE Healthcare), analyzed by SDS-PAGE, and pooled. Purified BG505-sCD4-17b-8ANC195 and B41-sCD4-21c-8ANC195 complexes were diluted to 200 μg/mL and 350 μg/mL in TBS, respectively, and vitrified in liquid ethane using a Mark IV Vitrobot (FEI). Sample grids were prepared by adding 3 μL of complex to glow discharged 400 Mesh Quantifoil R1.2/1.3 copper grids.

#### BG505-sCD4-17b-8ANC195 Complex Cryo-EM Data Collection and Processing

Images were recorded using SerialEM (Mastronarde, 2005) on a Titan Krios electron microscope equipped with Gatan K2 Summit direct detector. Exposures (15 s) were divided into 50 subframes with a dose rate of 3 electrons⋅pixel^−1^⋅subframe^−1^. After binning by two, each image was 4k×4k and 1.31 Å per pixel. A total of 3454 movies were collected using a 1-2.5 μm defocus range. The micrographs were motion corrected using MotionCor2 (Zheng et al., 2017) (Figure S1A) and contrast transfer function (CTF) estimations were calculated using CTFFIND4 (Rohou and Grigorieff, 2015) (Figure S1B). 2215 micrographs with CTF fitting beyond 4 Å were selected for automated particle picking using EMAN 2.2 (Ludtke et al., 2017). Subsequent steps were performed using RELION2 (Kimanius et al., 2016). A total of 548,229 particles were picked and sorted using initial 2D classifications. 213,791 particles from good 2D classes were selected for another round of 2D classification, which generated 195,755 “good” particles (Figure S1C). For 3D classification, an intermediate resolution cryo-EM structure of the BG505-sCD4-17b-8ANC195 complex (EMDB 8407) (Wang et al., 2016a) was low-pass filtered to 60 Å to serve as the reference, and then 195,755 particles were classified into 10 different 3D classes assuming only C1 symmetry (Figure S1C). Using 143,099 particles from six 3D classes as input for 3D refinement with C3 symmetry resulted in the highest resolution (4.68 Å) (Figure S1C). These particles were movie refined and polished before the final 3D refinement step, during which the Fab C_H_C_L_ and sCD4 D2 domains were masked out. After post-processing, the final resolution estimated by the gold-standard FSC (Scheres and Chen, 2012) was 3.54 Å (Figures S1C and S3A).

#### B41-sCD4-21c-8ANC195 Complex Cryo-EM Data Collection and Processing

Images were recorded using SerialEM (Mastronarde, 2005) on a Titan Krios electron microscope equipped with Gatan K2 Summit direct detector. Exposures (10 s) were divided into 50 subframes with a dose rate of 2 electrons⋅pixel^−1^⋅subframe^−1^. After binning by two, each image was 4k×4k and 1.31 Å per pixel. A total of 2531 movies were collected using a 1.7-3.5 μm defocus range. Subsequent steps were performed in RELION2 (Kimanius et al., 2016). The micrographs were motion corrected using MotionCor2 (Zheng et al., 2017) (Figure S2A) and CTF estimations were calculated using CTFFIND4 (Rohou and Grigorieff, 2015) (Figure S2B). Single particles were manually selected in Relion (∼1000 particles) and used to create an initial particle stack that was 2D classified to generate a template for autopicking in Relion. Reference free, 2D classification of 474645 auto-picked particles was performed, and 346264 particles corresponding to class averages of the complex were selected (Figure S2C). For 3D classification, a model structure of the Env-sCD4-21c-8ANC195 complex was generated by replacing 17b Fab with 21c Fab in the partially-open BG505-sCD4-17b-8ANC195 structure (PDB: 5THR) (Wang et al., 2016a). The 21c Fab binding angle was determined by aligning gp120s from the gp120-sCD4-21c crystal structure (PDB: 3LQA) (Diskin et al., 2010) and the BG505-sCD4-17b-8ANC195 structure. The modeled structure was low-pass filtered to 60 Å to serve as the initial 3D reference, and then 346264 particles were classified into 4 different 3D classes, during which C3 symmetry was assumed and the Fab C_H_C_L_ domains were masked out (Figure S2C). Using 305049 particles from three 3D classes as input for 3D refinement with C3 symmetry resulted in the highest resolution (4.39 Å) (Figure S2C). These particles were movie refined and polished before the final 3D refinement step. After post-processing, the final resolution estimated by the gold-standard FSC (Scheres and Chen, 2012) was 4.06 Å (Figure S3A). Regions distal to the gp120-gp41 core, including 21c, sCD4, and the displaced V1V2, were further classified and refined in Relion to enable de novo model building (See Figures S2C and S3 for details).

#### Cryo-EM Model Building

Coordinates for individual components of the 3.54 Å BG505-sCD4-17b-8ANC195 cryoEM structure were docked into their corresponding density regions using UCSF Chimera (Pettersen et al., 2004). The following coordinate files were used for docking: gp120 from a B41-sCD4-17b complex structure in an open conformation (PDB: 5VN3), gp41 from the same structure, sCD4 D1 domain (PDB: 2NXY), 17b Fab (PDB: 2NXY) (Zhou et al., 2007), 8ANC195 Fab (PDB: 4P9M) (Scharf et al., 2014). The docked coordinates were used as the template for RosettaCM (Song et al., 2013) to build an initial model of BG505 Env, after which the model were manually modified in Coot (Emsley and Cowtan, 2004) to remove residues that did not fit into density or clashed with other components. Coordinates for BG505 plus sCD4 and the Fabs were refined by applying C3 symmetry using Rosetta (Tange, 2011, Wang et al., 2016b). Coordinates for ordered N-linked glycans from PDB: 5T3X and PDB: 5VN3 Env structures (Gristick et al., 2016, Ozorowski et al., 2017) were fit separately as rigid bodies at potential N-linked glycosylation sites (PNGSs) at which EM density was apparent (Figure S4B), and glycan rings outside of EM density were removed. The entire complex was refined using phenix.real_space_refine with secondary structure restraints for protein and geometric restraints for protein and N-glycan residues (Adams et al., 2010, Afonine et al., 2018, Agirre et al., 2015, Gristick et al., 2017) (Table S1).

An initial model was generated for the 4.06 Å B41-sCD4-21c-8ANC195 cryoEM map by docking coordinates of individual components into their corresponding density regions with UCSF Chimera (Pettersen et al., 2004). Coordinates for gp120-gp41, sCD4 D1 domain, 21c Fab, and 8ANC195 Fab were obtained from PDB: 5VN3, 2NXY, 3LMJ, and 4P9M, respectively. Docked coordinates were initially rigid-body refined in Coot (Emsley and Cowtan, 2004), followed by several cycles of refinement in Phenix (Adams et al., 2010, Afonine et al., 2018) and manual rebuilding in Coot. Coordinates for ordered N-linked glycans from PDB: 5T3X and PDB: 5VN3 Env structures (Gristick et al., 2016, Ozorowski et al., 2017) were fit separately as rigid bodies at potential N-linked glycosylation sites (PNGSs) where EM densities for N-glycans were apparent (Figure S4B), and subsequently trimmed and refined in Coot to fit the cryo-EM map.

Density corresponding to the previously-uncharacterized V1V2 residues 129-195 was interpreted in the B41-sCD4-21c-8ANC195 structure as follows. After focus classification and refinement on the 21c, sCD4, V1V2 region (Figures S2C and S3), we identified density characteristic of a 2-stranded anti-parallel β-sheet that resembled the strand B–connecting loop–strand C conformation in known V1V2 structures (Figure S4G). An initial polyalanine model for strand B-connecting loop-strand C residues 154-174 was generated using coordinates from a closed BG505 crystal structure (PDB: 5T3Z) and manually docked into density (Figure S4G). The remaining portions of V1V2 could be placed by first building residues to continue the β2 strand into strand A and residues preceding β3 as strand D. We next found density compatible with a β-strand that could form a β-sheet interaction with strand D and assigned that as the V2 strand. Remaining density appearing to connect strands A and B via a long loop were left unmodeled due to uncertainty of the sequence register. β-strand restraints were applied to strands A-D and V2 throughout refinement in both Coot and Phenix. The entire complex was refined using phenix.real_space_refine with secondary structure restraints for protein and geometric restraints for protein and N-glycan residues (Adams et al., 2010, Afonine et al., 2018, Agirre et al., 2015, Gristick et al., 2017) (Table S2).

### Data and Software Availability

Cryo-EM reconstructions of the BG505-sCD4-17b-8ANC195 and B41-sCD4-21c-8ANC195 complexes have been deposited in the Electron Microscopy Data Bank under the accession numbers 7516 and 9038, respectively. Coordinates for atomic models of the BG505-sCD4-17b-8ANC195 and B41-sCD4-21c-8ANC195 complexes have been deposited in the Protein Data Bank under the accession numbers 6CM3 and 6EDU, respectively.

## Supplemental Information

Document S1. Figures S1–S7 and Tables S1–S3

Document S2. Article plus Supplemental Information
