# Supplementary material for: Partially Open HIV-1 Envelope Structures Exhibit Conformational Changes Relevant for Coreceptor Binding and Fusion
Source: Cell Host Microbe. 2018 Oct 10;24(4):579–592.e4. doi: 10.1016/j.chom.2018.09.003 (PMC6185872; doi:10.1016/j.chom.2018.09.003)
Supplement: Document S1. Figures S1–S7 and Tables S1–S3 [file mmc1.pdf]

Cell Host & Microbe, Volume *24*

Supplemental Information

Partially Open HIV-1 Envelope Structures Exhibit Conformational Changes Relevant for Coreceptor Binding and Fusion

Haoqing Wang, Christopher O. Barnes, Zhi Yang, Michel C. Nussenzweig, and Pamela J. Bjorkman

Supplemental Figures and legends

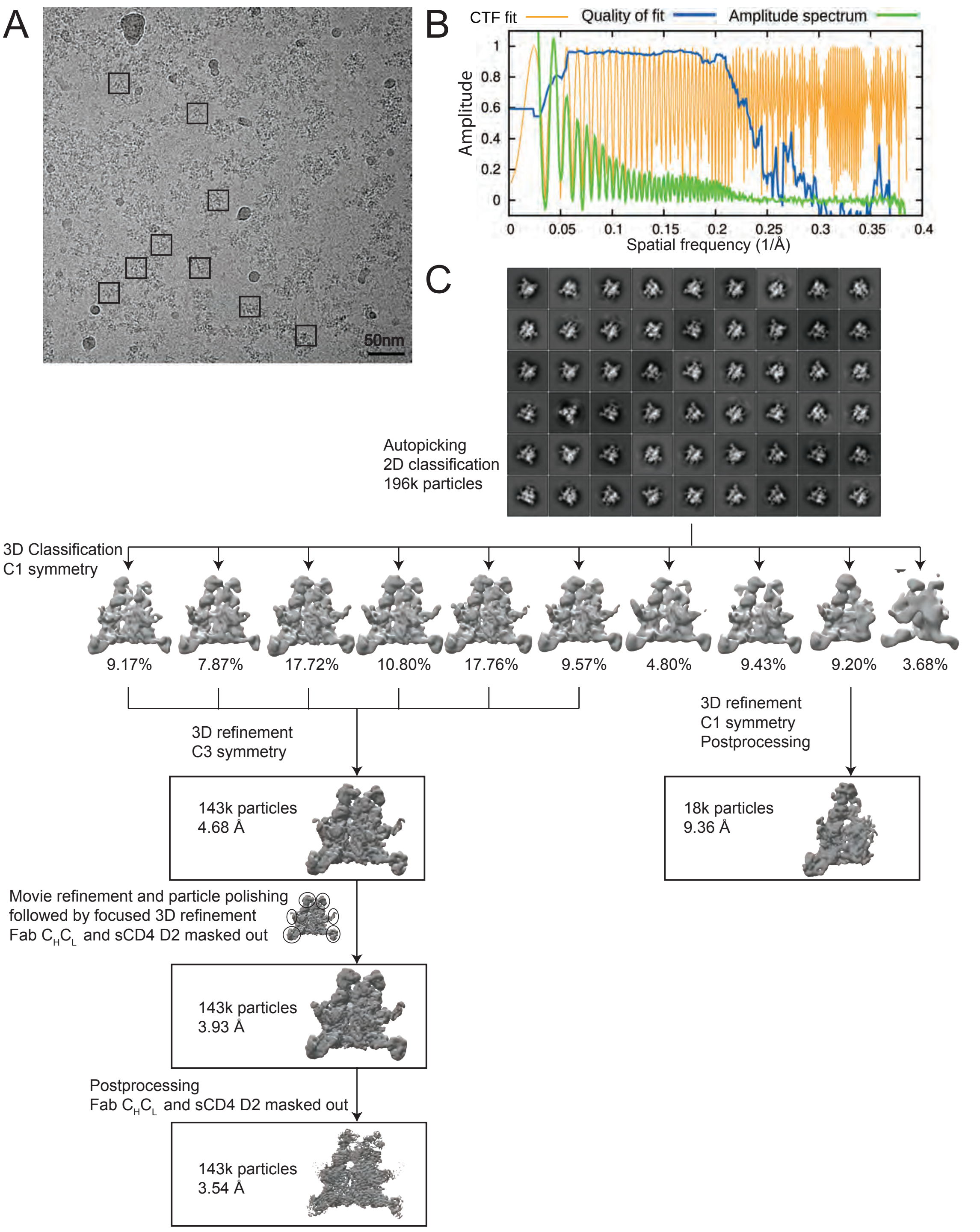

Figure S1. Data processing for the BG505-sCD4-17b-8ANC195 complex structure, related to Figure 1.

(**A**) Representative motion corrected and dose-weighted micrograph of BG505-sCD4-17b-8ANC195 complex (examples of individual particles in boxes). Scale bar of 50nm is shown. The defocus value was ~2.3 μm underfocus. (**B**) CTF fitting of the micrograph shown in (A) using CTFFIND4 (Rohou and Grigorieff, 2015), showing a good fit to 3.3 Å. (**C**) Data processing scheme. 2D classification of autopicked particles resulted in 196k "good" particles that were 3D classified using a 60 Å low-pass-filtered cryo-EM structure (EMDB 8407) as the reference model. The 143k particles from the best six 3D classes were refined to 4.68 Å resolution using C3 symmetry. The particles in the 4.68 Å reconstruction were further movie refined and polished using RELION (Scheres, 2012), and then refined with the Fab C_HC_L domains and sCD4 D2 domains masked out. The final post-processing step generated a 3.54 Å structure with three bound sCD4 plus three 17b and three 8ANC195 Fabs per Env trimer. One 3D class with 18k particles with two sCD4, two 17b, and two 8ANC195 bound to each Env trimer was refined with C1 symmetry, resulting in a 9.36 Å structure.

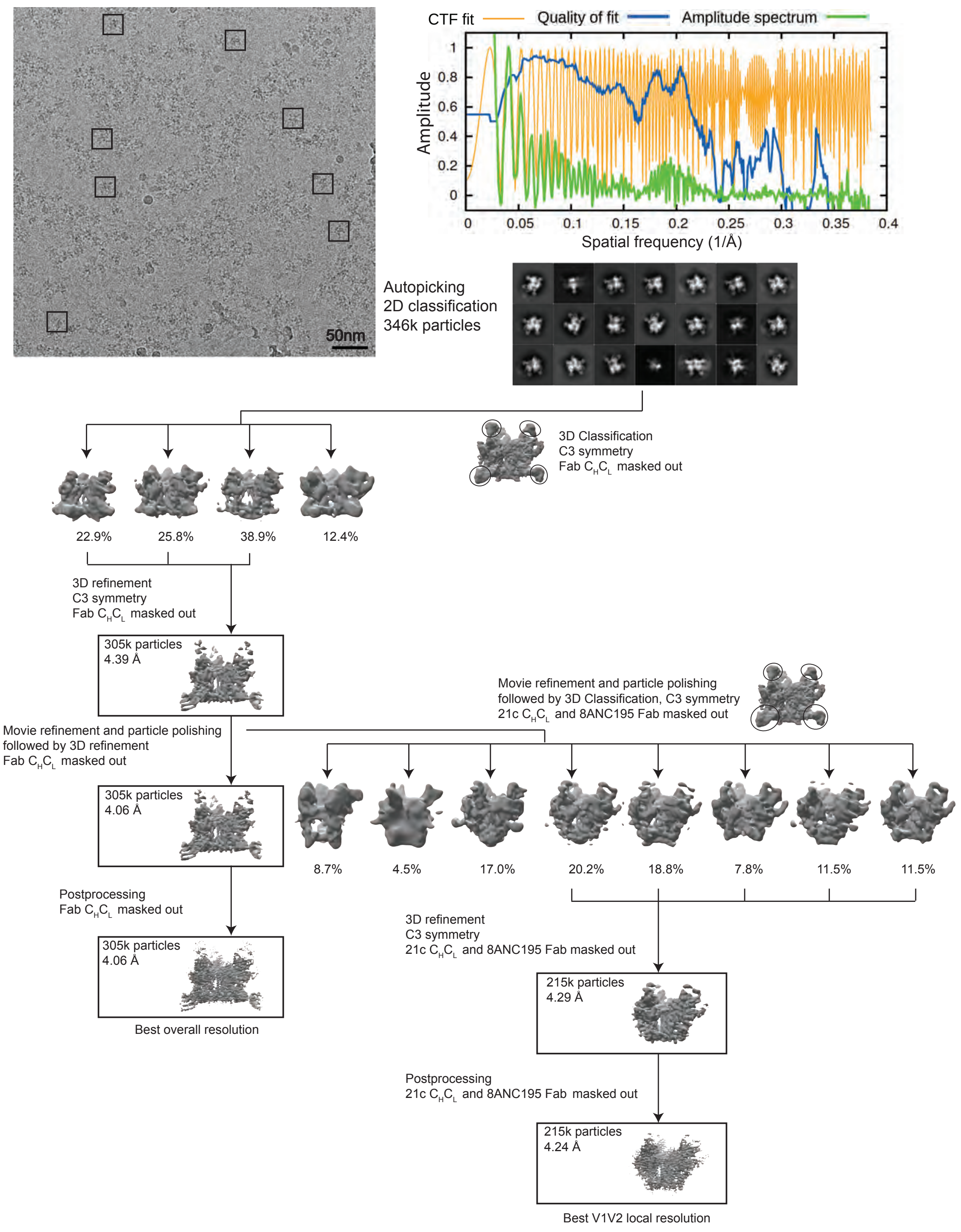

Figure S2. Data processing for the B41-sCD4-21c-8ANC195 complex structure, related to Figure 1.

(**A**) Representative micrograph with examples of B41-sCD4-21c-8ANC195 complex in boxes. The defocus value was ~2.6 μm underfocus. (**B**) CTF fitting of the left micrograph using CTFFIND4 (Rohou and Grigorieff, 2015) showing a good fit to 4.7 Å. (**C**) Data processing scheme. 2D classification of autopicked particles resulted in 346k "good" particles that were 3D classified into four 3D classes using a 60 Å low-pass-filtered reference model assuming C3 symmetry. The reference model was made by replacing 17b Fab with 21c Fab in partially-open BG505-sCD4-17b-8ANC195 structure (PDB 5THR). The 21c Fab binding angle was modeled using a gp120-sCD4-21c crystal structure (PDB 3LQA). During 3D classification and the following refinement steps the Fab C_HC_L domains were masked out. The 305k particles from the best three 3D classes were refined to 4.39 Å resolution. The particles in the 4.39 Å reconstruction were further movie refined and polished using RELION (Scheres, 2012), and then refined to 4.06 Å. The final post-processing step generated a 4.06 Å structure with three bound sCD4 plus three 21c and three 8ANC195 Fabs per Env trimer. After movie refinement and particle polishing we performed another round of 3D classification, during which the 21c C_HC_L domains and the 8ANC195 Fabs were masked out, which improved the local resolution in the vicinity of V1V2. 305k particles were classified into 10 different classes assuming C3 symmetry, and 5 classes with obvious V1V2 densities were selected for model building of the displaced V1V2. After post-processing, the final resolution was 4.24 Å.

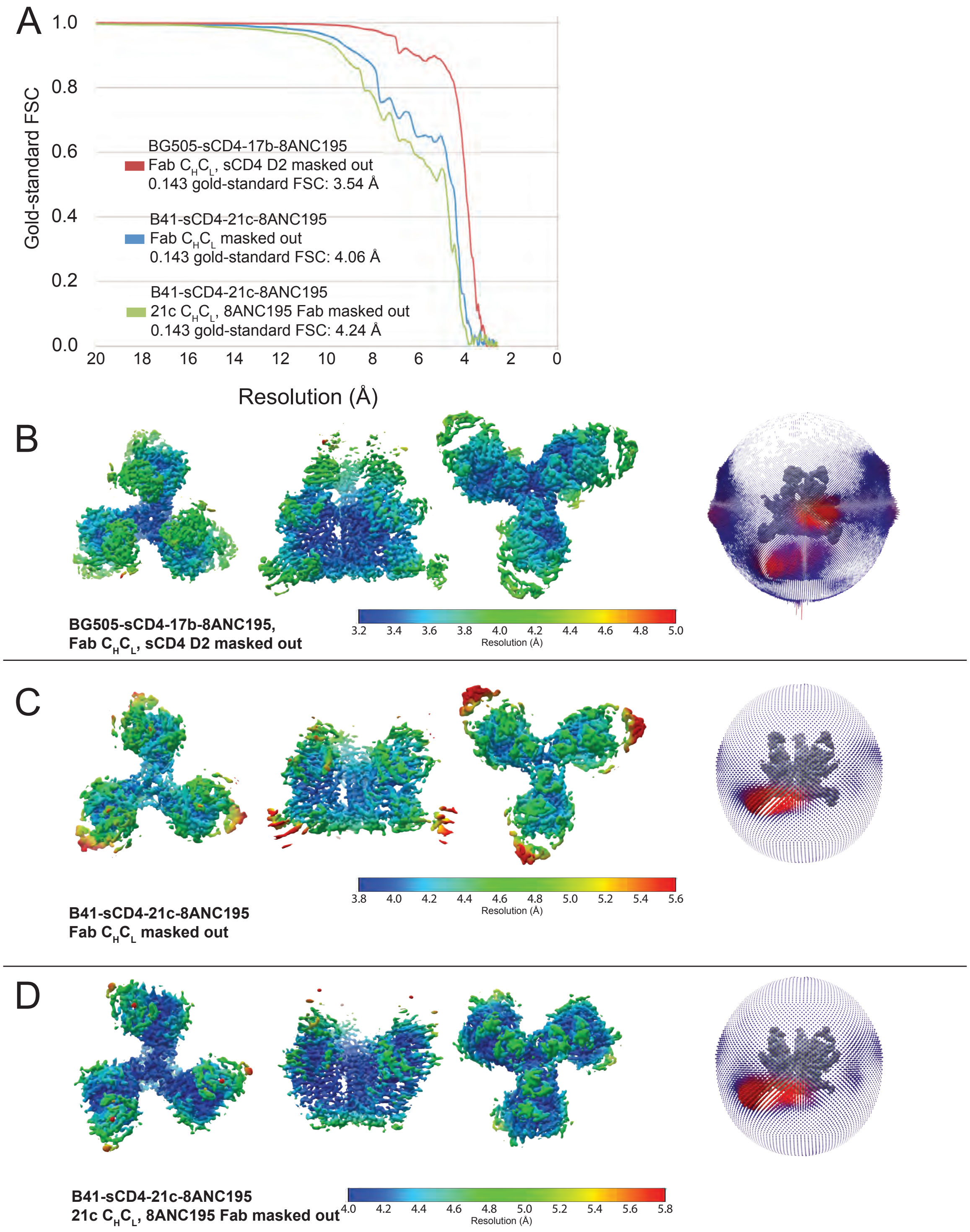

Figure S3. Validation of the BG505-sCD4-17b-8ANC195 and B41-sCD4-21c-8ANC195 complex structures, related to Figure 1.

(**A**) Gold-standard FSC of 3.54 Å BG505-sCD4-17b-8ANC195 structure, 4.06 Å B41-sCD4-21c-8ANC195 structure, and 4.24 Å B41-sCD4-21c structure in which 8ANC195 Fabs were masked out. (**B**) Local resolution estimation and orientation distribution for the 3.54 Å BG505-sCD4-17b-8ANC195 structure. (**C**) Local resolution estimation and orientation distribution for the 4.06 Å B41-sCD4-21c-8ANC195 structure. (**D**) Local resolution estimation and orientation distribution for the 4.24 Å B41-sCD4-21c structure.

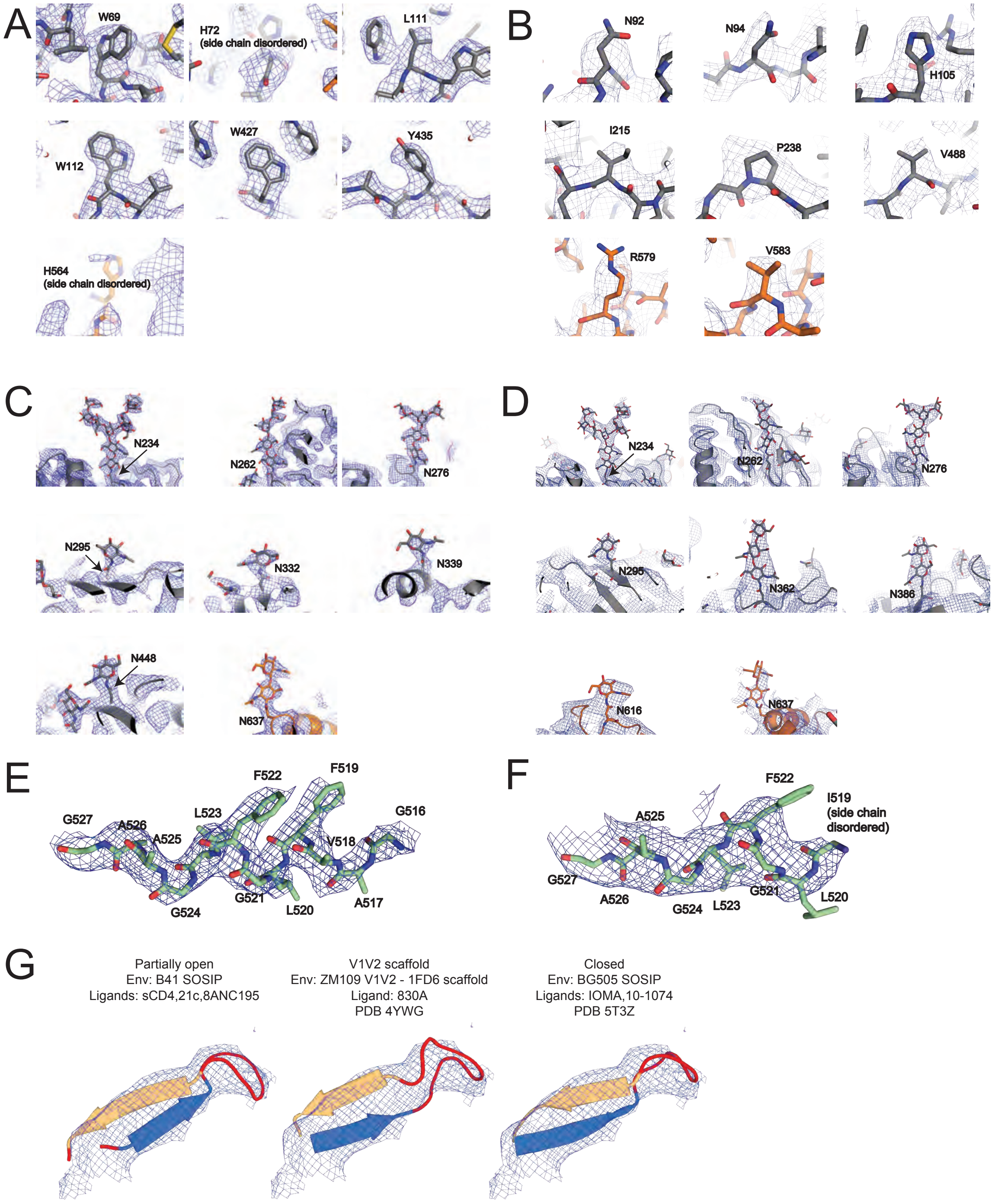

Figure S4. Close-up views of cryo-EM maps, related to Figures 1 and 4.

Close-up views of selected density regions in the 3.54 Å BG505-sCD4-17b-8ANC195 and 4.24 Å B41-sCD4-21c-8ANC195 structures. Maps were contoured at 10.5 σ (0.0571 e/Å^3) for the BG505-sCD4-17b-8ANC195 structure (**A, C, E**) and 9.0 σ (0.0305 e/Å^3) for the B41-sCD4-21c-8ANC195 structure (**B, D, F, G**). (**A**) Cryo-EM densities of conserved residues shown in **Figure 4A** in the partially-open BG505-sCD4-17b-8ANC195 structure. (**B**) Cryo-EM densities of residues in the partially-open B41-sCD4-21c-8ANC195 structure. (**C, D**) Cryo-EM densities of N-linked glycans attached to the indicated Asn residues for BG505-sCD4-17b-8ANC195 (**C**) and B41-sCD4-21c-8ANC195 (**D**) structures. (**E, F**) Cryo-EM densities of fusion peptide regions in BG505-sCD4-17b-8ANC195 (**E**) and B41-sCD4-21c-8ANC195 (**F**) structures. (**G**) Cryo-EM density of the strand B–connecting loop–strand C region of the displaced V1V2 in the B41-sCD4-21c-8ANC195 structure. The density is shown as fit by strand B–connecting loop–strand C coordinates from the B41-sCD4-21c-8ANC195 structure (left), from a V1V2 scaffold structure (middle), and from a closed Env structure (right), demonstrating a consistent strand B–connecting loop–strand C geometry among different structures.

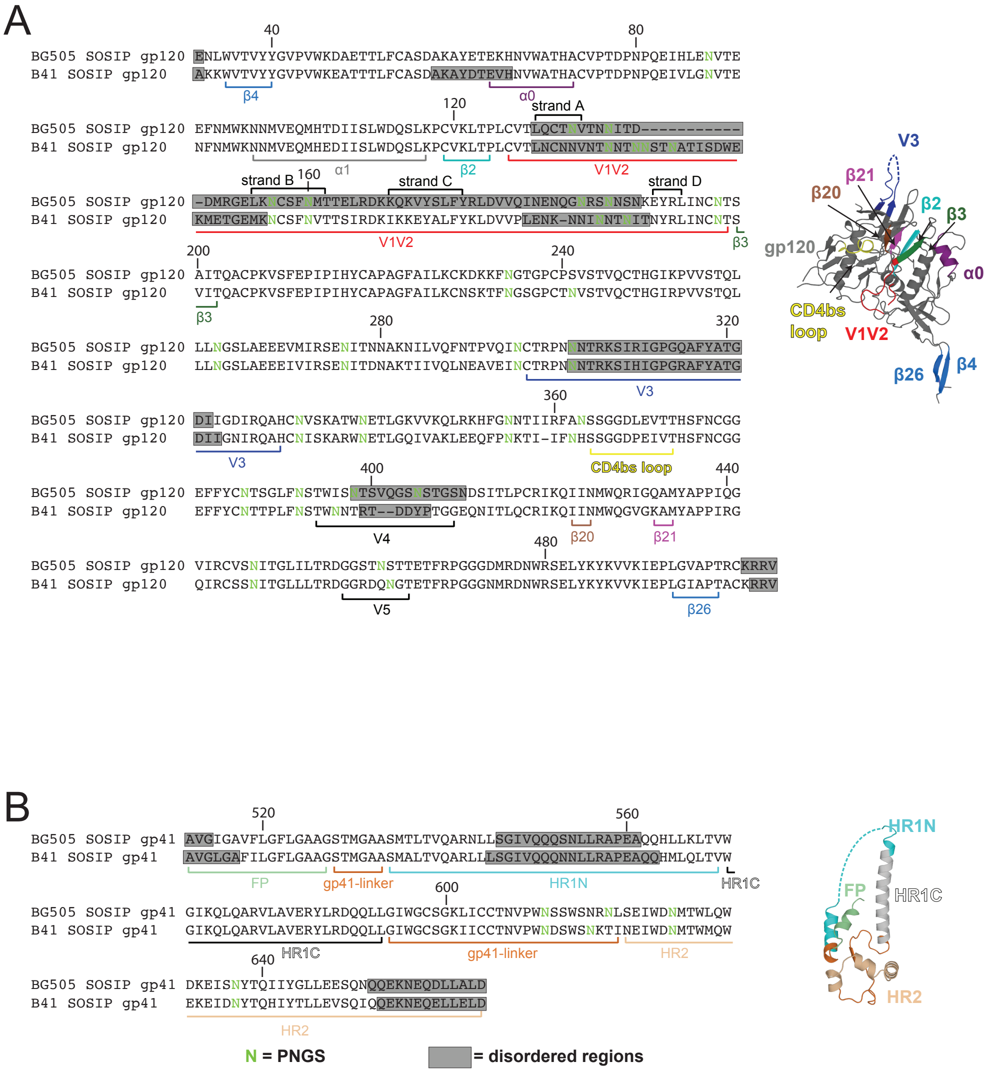

Figure S5. Env sequence alignments and definitions of secondary structural elements, related to Figure 1, 2, and 3.

(A) Left: gp120 sequence alignment of BG505 SOSIP and B41 SOSIP. Potential N-linked glycosylation sites (PNGSs) are highlighted in green. Disordered regions in the BG505-sCD4-17b-8ANC195 and B41-sCD4-21c-8ANC195 structures are indicated by a grey background. Secondary structure elements are highlighted below the sequence alignments. Right: gp120 structure from the 3.54 Å BG505-sCD4-17b-8ANC195 complex structure. Secondary structure elements are highlighted in the same colors as in the sequence alignment. (**B**) gp41 sequence alignment and structure as in (**A**).

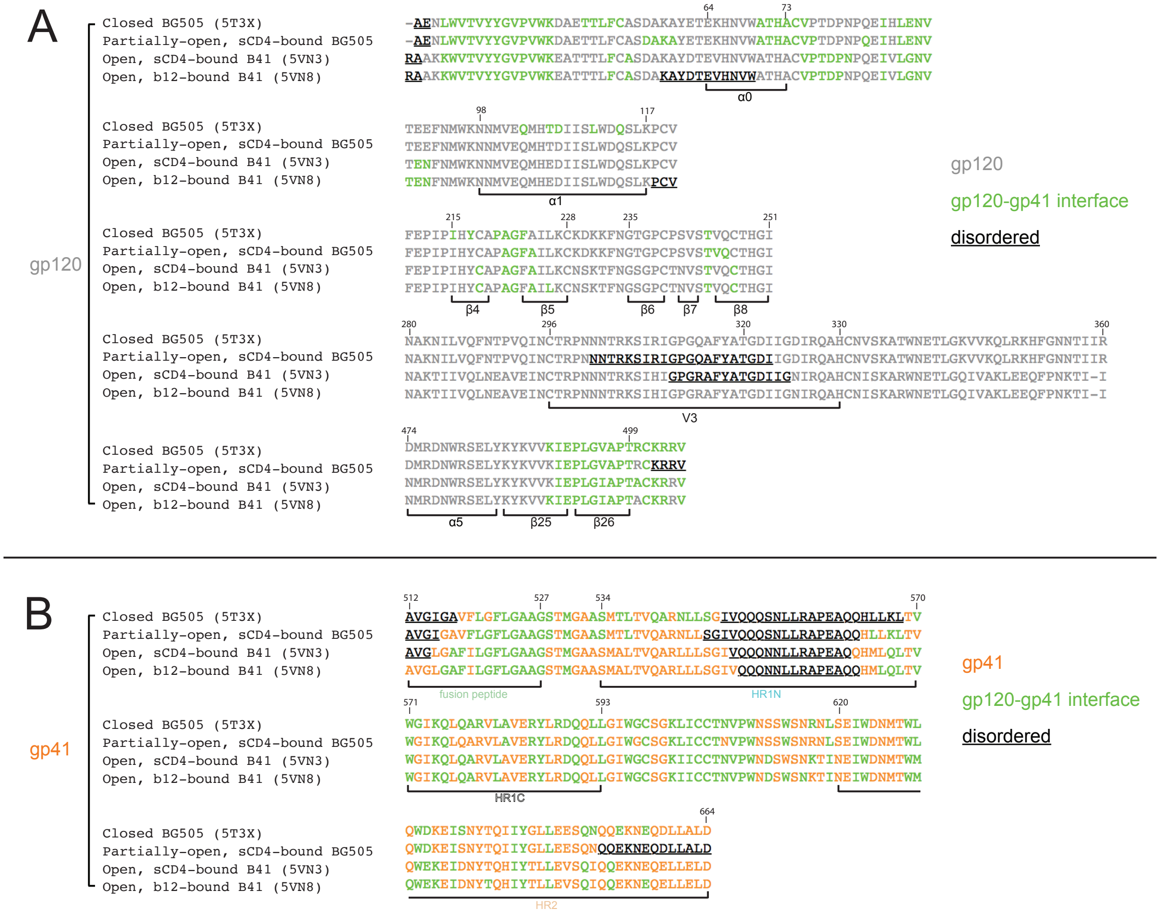

Figure S6. gp120-gp41 interface residues, related to Figure 3.

(**A**) gp120 sequence alignment for partially-open BG505-sCD4-17b-8ANC195 structure (this study), open Envs (PDBs 5VN3 and 5VN8), and closed Envs (PDB 5T3X). Disordered regions are indicated by an underscore. gp120-gp41 interface residues (defined as the gp120 and gp41 residues that include atoms within 5 Å of each other) are highlighted in green. Secondary structure elements are labeled below the sequence alignment. (B) gp41 sequence alignment as in (**A**).

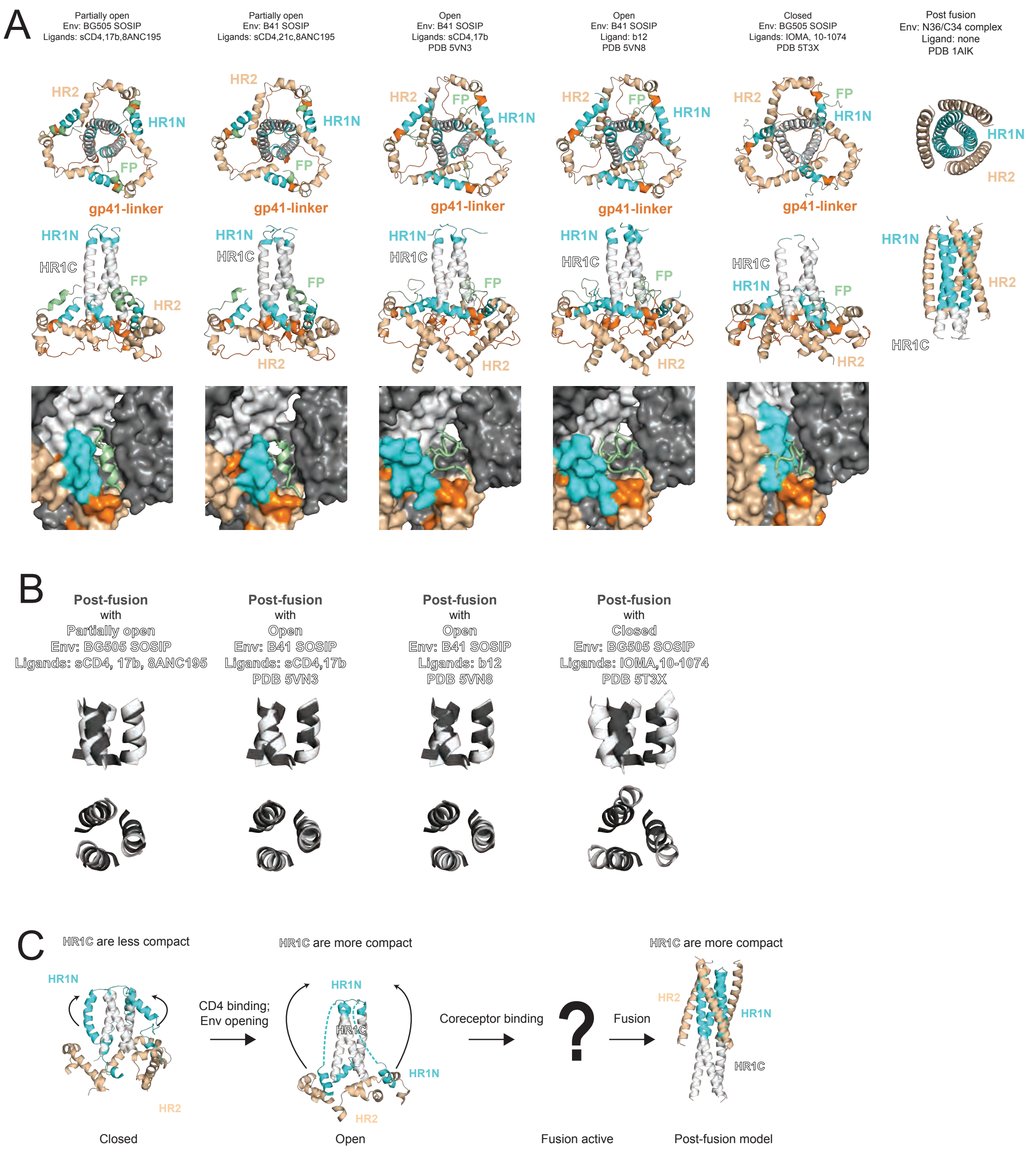

Figure S7. gp41 structures in different conformations, related to Figure 3.

(A) Trimeric gp41 structures from partially-open Envs **(**BG505-sCD4-17b-8ANC195 and B41-sCD4-21c-8ANC195**)**, open Envs (PDBs 5VN3 and 5VN8), closed Envs (PDB 5T3X), and a post-fusion gp41 core structure (PDB 1AIK). Upper panel: top views of gp41; middle panel: side views gp41; bottom panel: zoom-in view of fusion peptide residues (cartoon representation) overlaid on surface representations of Env

trimer structures. **(B)** Superimpositions of post-fusion gp41 HR1 structure with gp41 HR1 structures from Env in different conformations. Post-fusion gp41 HR1s are shown in black while gp41 HR1s from Env shown in white. (C) Proposed model of gp41 conformational changes during viral infection.

Supplemental Tables

Table S1. Data collection and model statistics for the BG505-sCD4-17b-8ANC195 complex structure, related to Figure 1.

Data collection/processing	
Microscope	Titan Krios
Voltage	300kV
Camera	Gatan K2 Summit
Camera mode	Super-resolution
Defocus range	1.0-2.5
Exposure time	15
Dosage rate	1.75 electrons/Å^2/subframe
Magnified pixel size	1.31 Å
Total dose	87.5 electrons/Å^2
**Reconstruction**	
Software	Relion-2.0
Symmetry	C3
Particles refined	143k
Final Resolution(Å)	3.54
Map sharpening B-factor (Å^2)	-95.4595
**Model Statistics**	
Mask CC	0.7744
Volume CC	0.759
Peak CC	0.602
All-atom clashscore	5.41
Ramachandran plot:	
outliers	0.00%
allowed	6.78%
favored	93.22%
Rotamer outliers	0.11%
C-beta deviations	0.00%
RMSD (bonds) (Å)	0.00
RMSD (angles) (°)	0.87
EMRinger Score	2.32

Table S2. Data collection and model statistics for the B41-sCD4-21c-8ANC195 complex structure, related to Figure 1.

Data collection/processing	
Microscope	Titan Krios
Voltage	300kV
Camera	Gatan K2 Summit
Camera mode	Super-resolution
Defocus range	1.7-3.5
Exposure time	10
Dosage rate	1.16 electrons/Å^2/subframe
Magnified pixel size	1.31 Å
Total dose	58.28 electrons/Å^2
**Reconstruction**	
Software	Relion-2.1
Symmetry	C3
Particles refined	215k
Final Resolution(Å)	4.24
Map sharpening B-factor (Å^2)	-220.00
**Model Statistics**	
Mask CC	0.808
Volume CC	0.805
Peak CC	0.550
All-atom clashscore	8.68
Ramachandran plot:	
outliers	0.19%
allowed	11.60%
favored	88.21%
Rotamer outliers	0.34%
C-beta deviations	0.00%
RMSD (bonds) (Å)	0.01
RMSD (angles) (°)	1.12
EMRinger Score	1.06

Table S3. Distances^ between key residues in different Env trimer conformations, related to Figure 2.

Trimer state	Ligand	Trimer type	Method	EMDB/ PBD ID	Resolution (Å)	Distance between V1V2 base (P124) (Å)	DIstance between CD4bs (D368) (Å)	Distance between V3 base (H330) (Å)
closed	8ANC195	BG505 SOSIP.664	X-ray	5CJX	3.58	14	54	68
closed	PGT122, 35O22	BG505 SOSIP.664	X-ray	4TVP	3.5	15	55	69
closed	PGT122	BG505 SOSIP.664	X-ray	4NCO	4.7	14	56	70
closed	3H+109L, 35O22	BG505 SOSIP.664	X-ray	5CEZ	3	14	56	69
closed	IOMA, 35O22	BG505 SOSIP.664	X-ray	5T3Z	3.5	14	54	69
closed	PGT151	JR-FL EnvΔCT	cryo-EM	5FUU	4.19	16	56	69
					**Average Distance**	**15±1**	**55±1**	**69±1**
partially open	sCD4, 17b, 8ANC195	BG505 SOSIP.664	cryo-EM	5THR	8.9	68	78	73
partially open	sCD4, 17b, 8ANC195	BG505 SOSIP.664	cryo-EM	this study	3.54	67	79	76
partially open	sCD4, 21c, 8ANC195	B41 SOSIP.664	cryo-EM	this study	4.06	69	79	73
					**Average Distance**	**68±1**	**79±1**	**74±2**
open	sCD4, 17b	B41 SOSIP.664	cryo-EM	5VN3	3.7	79	84	73
open	b12	B41 SOSIP.664	cryo-EM	5VN8	3.6	69	84	75

^Inter-subunit distances were measured for indicated $C\alpha$ atom positions in PyMol.
